# Insights into the evolution and domain structure of ataxin-2 proteins across eukaryotes

**DOI:** 10.1186/1756-0500-7-453

**Published:** 2014-07-15

**Authors:** Domingo Jiménez-López, Plinio Guzmán

**Affiliations:** 1 Departamento de Ingeniería Genética, Centro de Investigación y de Estudios Avanzados, Unidad Irapuato, Apartado Postal 629, Irapuato, Gto 36821, México

**Keywords:** Spinocerebellar ataxia type 2, Phylogenetic analysis, Polyglutamine expansions, PAM2, Poly(A)-binding protein, MLLE domain

## Abstract

**Background:**

Ataxin-2 is an evolutionarily conserved protein first identified in humans as responsible for spinocerebellar ataxia type 2 (SCA2). The molecular basis of SCA2 is the expansion of a polyglutamine tract in Ataxin-2, encoding a Lsm domain that may bind RNA and a PAM2 motif that enables interaction with the poly (A) binding protein. Although the association with SCA2 has been verified, a detailed molecular function for Ataxin-2 has not been established.

**Results:**

We have undertaken a survey of Ataxin-2 proteins across all eukaryotic domains. In eukaryotes, except for vertebrates and land plants, a single ortholog was identified. Notably, with the exception of birds, two Ataxin-2 genes exist in vertebrates. Expansion was observed in land plants and a novel class lacking the LsmAD domain was identified. Large polyQ tracts appear limited to primates and insects of the orders Hymenoptera and Diptera. A common feature across Ataxin-2 orthologs is the presence of proline-rich motifs, formerly described in the human protein.

**Conclusion:**

Our analysis provides valuable information on the evolution and domain structure of Ataxin-2 proteins. Proline-rich motifs that may mediate protein interactions are widespread in Ataxin-2 proteins, but expansion of polyglutamine tracts associated with spinocerebellar ataxia type 2, is present only in primates, as well as some insects. Our analysis of Ataxin-2 proteins provides also a source to examine orthologs in a number of different species.

## Background

Gene expression in eukaryotes is regulated from the modulation of chromatic structure to the synthesis and assemblage of proteins. Accurate control at each stage is fundamental for the proper functioning of the cell. The Ataxin-2 protein has been implicated in the broad modulation of local mRNA translation. Normal human Ataxin-2 alleles contain up to 30 glutamine repeats at the amino-terminus, with 22 repeats being the average for most populations. An expansion of the polyglutamine (polyQ) tract in Ataxin-2 was identified as responsible for spinocerebellar ataxia type 2 (SCA2), a progressive neurodegenerative disease. SCA2 results from polyQ expansion over a certain threshold. Repeats of 32 and more cause SCA2 with a characteristic degeneration of cerebellar Purkinje cells [1].

Domain structure of the Ataxin-2 protein provides information regarding its molecular function in RNA metabolism. It encodes a Like RNA splicing domain Sm1 and Sm2 (Lsm) and a Like-Sm-associated domain (LsmAD) at the amino-terminal region. The Lsm domain likely binds to RNA and the LsmAD domain includes a clathrin-mediated trans-Golgi signal [2-4]. It has also been established that the DEAD/H-Box RNA Helicase DDX6 binds to the LSm/LSmAD domain of Ataxin-2. DDX6 is a component of stress granules, ribo-nucleoprotein particle aggregates that carry translationally repressed mRNAs [5]. Ataxin-2 also carries a poly (A)-binding protein interacting motif 2 (PAM2) at the carboxy-terminus that mediates the interaction with the MLLE domain, of the cytoplasmic poly (A)-binding protein (PABP). MLLE (MademoiseLLE) also known as CTC or PABC is named after a conserved KITGMLLE signature sequence and found at the C-terminus of PABP. PABP is an evolutionarily conserved RNA-binding protein that includes four RNA recognition domains. PABP binds to the poly (A) tail of mRNAs and is essential for translation initiation and mRNA decay. The MLLE domain of PABP binds proteins, such as Ataxin-2, that contain a PAM-2 motif. PABP is also a component of the stress granules [6-8]. The eukaryotic releasing factor 3 (eRF3) that mediates deadenylation coupled to translation termination contains an unusual PAM2 motif. eRF3 possesses a tandem reiteration of overlapping PAM2 sequences that endow this domain with distinctive functional features [9,10].

Ataxin-2 is evolutionarily conserved, and its function has been linked to stress granules and translation regulation in model organisms besides humans [11-16]. Ataxin-2-like, a human paralog of Ataxin-2, has also been indentified. Ataxin-2-like is structurally similar to Ataxin-2, except that it does not contain the poly (Q) tract. Ataxin-2-like binds to DDX6 and PABP and is a component of stress granules, suggesting a functional overlap between the two paralogs [17].

We have previously predicted a set of MLLE-interacting proteins in the *C*TC-*I*nteracting *D*omain (CID) of plants. Among them, two *Arabidopsis thaliana* (CID3 and CID4) and one *Oriza sativa* (9630.m02659) Ataxin-2 orthologs were identified [18]. These putative orthologs encode Lsm, LsmAD, and a PAM2 motif that shows a tandem reiteration of two overlapping PAM2 sequences, similar to the PAM2 motif in eRF3. We relied on the accessibility of genome sequences and gene annotation from several complete genomes from evolutionarily divergent eukaryotes to provide detail information on the distribution, domain architecture and evolution of Ataxin-2 genes. Ataxin-2 genes seem to have arisen early after the origin of eukaryotes and their expansion and domain structure differ in different eukaryotic lineages. This study uncovered novel model systems useful to get insights on how this protein involved in RNA metabolism operates. We identified and performed our analysis on 216 putative Ataxin-2 orthologs from 127 species including plants, animals, fungi, and protists.

## Results and discussion

### Identification of ataxin-2 genes across eukaryotes

**Figure 1:**
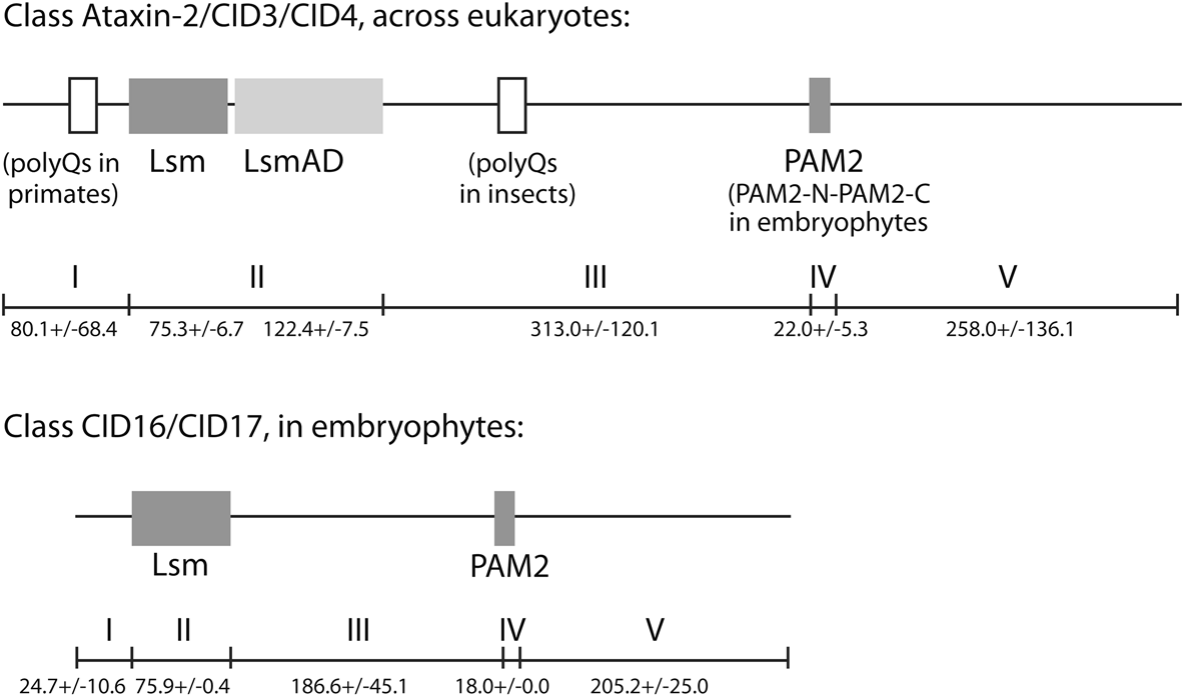
**A broad view of domain architecture of Ataxin-2 proteins.** A schematic representation of the canonical Ataxin-2 protein (Ataxin-2/CID3/CID4 class), and a novel class present in embryophytes (CID16/CID17 class). The five modules proposed for Ataxin-2 proteins are depicted. The numbers between the regions correspond to the mean value and standard deviation of each module in number of amino acid residues.

The domain architecture for Ataxin-2 is portrayed in Figure 1, which reveals Lsm and LsmAD domains in all orthologs described to date. We expanded an examination of the domain structure through a broad scale evolutionary study of Ataxia-2 proteins across genomes from plants, animals, fungi, and protists. We used the well conserved Lsm domain to search for putative orthologs using BLASTP based on the human Ataxin-2 protein and the *Arabidopsis thaliana* CID3 ortholog (see Material and methods). We previously identified CID3 and CID4 as two putative *A. thaliana* Ataxin-2 orthologs [18]. A collection of 216 sequences containing the Lsm domain was retrieved from 127 species: 56 animals, 40 plants, 15 fungi, and 16 protozoa. Putative orthologs were identified across all species, indicating that Ataxin-2 is an important element of core genes in eukaryotic genomes.

**Figure 2:**
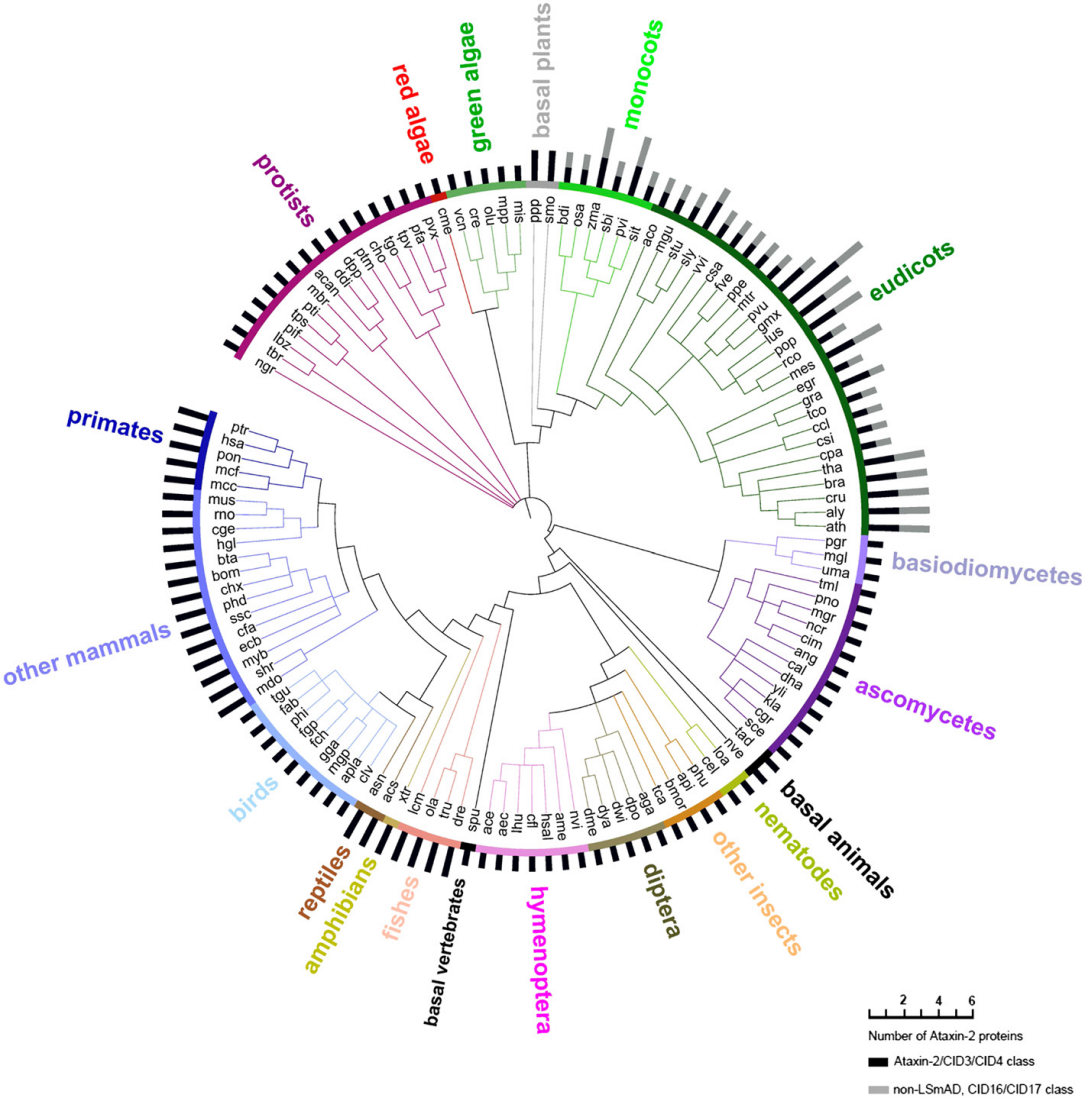
**Number of retrieved Ataxin-2 proteins in eukaryotes.** The phylogenetic relationship between thirty-eight vertebrates, eighteen invertebrates, fifteen fungi, sixteen protists and forty viridiplantae genomes is displayed in a circle. Relationships were adapted from the National Center of Biotechnology Information (NCBI) taxonomy server (http://www.ncbi.nlm.nih.gov/Taxonomy) [36]. The name of major group of organisms is displayed with a color code. The species abbreviations and the proteins are listed in Additional file 1.

A single Ataxin-2 gene was retrieved from protists, fungi and green algae (chlorophytes), one or two from animals, and two to six from land plants (embryophytes) (Figure 2 and Additional file 1). A noteworthy observation is that two genes were identified in vertebrates, with the exception of birds, only one gene was found in the nine avian genomes analyzed. All other animal species possessed one Ataxin-2 gene. Based on the fact that whole-genome duplication events occurred early in vertebrates, it is possible that the vertebrate lineage originally contained two Ataxin-2 copies, but that only one was maintained in avian genomes [19,20].

### CID16/CID17, a novel class of ataxin-2-like gene in embryophytes (land plants)

We inspected the orthologs for the presence of the LsmAD domain that is adjacent to Lsm. All othologs examined from animals, fungi, and protozoa encoded LsmAD domains, but in plants, where two othologs were present, only one was a *bona fide* Ataxin-2 protein, encoding Lsm and LsmAD domains, whereas the other ortholog lacked the LsmAD domain. Domain architecture inspection revealed that this new class of Ataxin-2-like proteins retained the PAM2-related sequences (see below). Based on our previous nomenclature of PABP-interacting proteins (*C*TC-*I*nteracting *D*omain [18]), we named the two *A. thaliana* genes *CID16* (At4g26990) and *CID17* (At5g54920), neither of which contained the LsmAD domain (Figure 2, dark and gray bars, respectively). An alignment of the canonical CID3/CID4 class and the CID16/CID17 from *A. thaliana* is shown in Additional file 2.

After inspecting forty plant genomes, at least one ortholog from each class of Ataxin-2 was identified in all angiosperm genomes examined. Variation in the number of orthologs correlated with genome duplication events that occurred during the evolution of land plants. Duplicated paralogs were observed in species that underwent recent or additional duplication events (*Linum usitatissimum*, *Populus trichocarpa*, *Glycine max*, *Brassica rapa*) [21-24], and is readily visible within the five species examined of the Brassicaceae (*A. thaliana*, *Arabidopsis lyrata*, *Capsella rubella*, *Thellungiella halophita* and *Brassica rapa*). The domain structure indicated that both of the two Ataxin-2 genes identified in the two basal angiosperms (the moss *Physcomitrella patens* and the lycopod *Selaginella moellendorffii*) correspond to the CID3/CID4 class, suggesting that the CID16/CID17 class originated early in the flowering plant lineage. This new class of Ataxin-2 genes is absent in other eukaryotes, and may be an indicator of neofunctionalization or subfunctionalization of Ataxin-2 genes in flowering plants. The Ataxin-2 protein has been mostly found in the Golgi apparatus, and deletion of the LsmAD domain affects its subcellular localization [25]. The LsmAD domain has also been implicated in the recruitment of the helicase DDX6, thus the absence of this domain in the CID16/CID17 class might confer novel subcellular distributions and functional properties Ataxin-2-related proteins.

Additionally, a truncated version of the CID3/CID4 class that do not contained the carboxy-terminal region was identified in *Zea mays* (GRMZM2G056773 encodes Lsm and LsmAD domains but not the region containing the PAM2 motif). Yet, *Zea mays* contains and additional full copy of the CID3/CID4 class of Ataxin-2 (GRMZM5G829738).

To assist in a comprehensive view of the domain architecture among Ataxin-2 orthologs, we divided a canonical Ataxin-2 protein five modules based on positional references to the Lsm and PAM2 domains (Figure 1). These five modules are located: (I) from the amino-terminus to the Lsm domain; (II) the Lsm and LsmAD domains; (III) between the LsmAD (or Lsm in the CID16/CID17 class) and the PAM2 domains; (IV) the PAM2 motif; (V) from the PAM2 motif to the carboxy-terminus. To estimate the average length of Ataxin-2 proteins, the mean values and standard deviations of each module was determined (Figure 1). Inspection of module size indicated that the average size of LSM, LsmAD and PAM2 motifs (modules II/III) is preserved across all eukaryotes and the size of modules I, III and V is highly variable (Figure 1).

### Phylogenetic distribution of ataxin-2 proteins in eukaryotes

**Figure 3:**
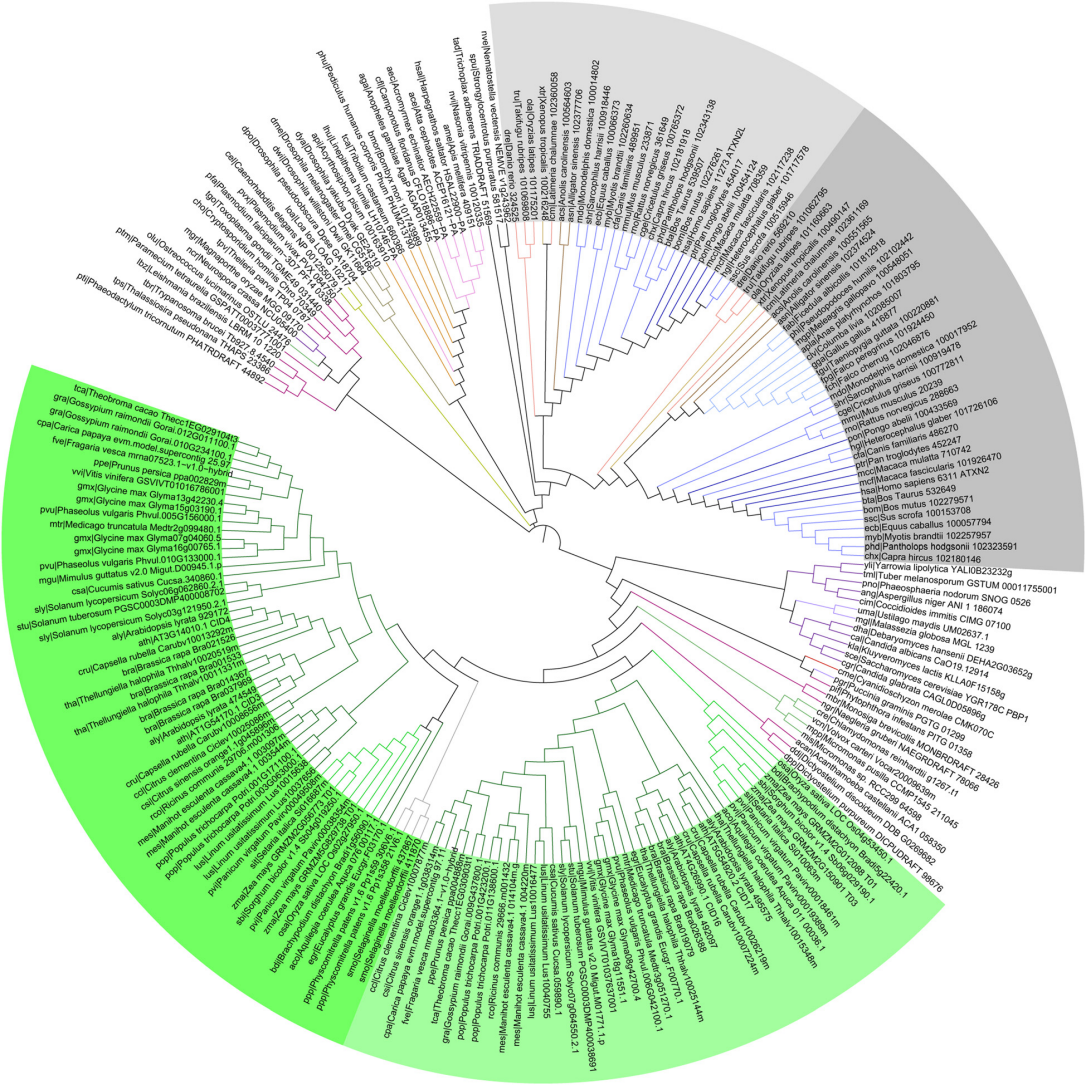
**Phylogeny of Ataxin-2 proteins.** The Lsm domain was used to obtain the tree. The topology was generated by the NJ method. Species and gene names are as mentioned in Additional file 1. Color codes of branches are as depicted in Figure 2. Vertebrate proteins are shadowed in gray tones (Ataxin-2, in dark gray and Ataxin-2-like in light gray) and plant proteins in green tones (CID3/CID4 class in dark green and CID16/CID17 class in light green). The rectangular phylogeny is shown in Additional file 4.

To obtain phylogenies from the 216 Ataxin-2 proteins with support for branch classification and conventional resolution of species, we compared trees based on complete protein sequences with trees based on the conserved Lsm domain, thus eliminating divergent regions (Figure 3). We generated and compared phylogenies generated with neighbor-joining (NJ), maximum-parsimony (MP) and maximum-likelihood (ML) methods (see Methods). The resulting trees had similar topologies, with plant and animal lineages grouped as separate clades. The tree based on the Lsm domain generated with a NJ method showed less events of taxonomic incongruence in most of the clades than the phylogenies obtained through ML and MP. This tree is shown in Figure 3. The trees generated with MP and ML methods are shown in Additional file 3 (trees based on complete protein sequences are not shown).

Sequences from the placozoan *Trichoplax adhaerens TRIADDRAFT 51569*, and the cnidarian *Nematostella vectensis* NEMVE v1g243962 (sea anemone), two basal eukaryote lineages, exhibited an incongruent taxonomic association since they appeared to be basal to vertebrates only; misplacement of basal animal species were also observed in phylogenetic analysis of RING finger ligases families across eukaryotes [26]. The echinoderm *Strongylocentrotus purpuratus* 581517(sea urchin), which is basal to vertebrates, was properly placed. Sequences belonging to red algae and four from the green algae (except *Ostreococcus lucimarinus* OSTLU 24476), occurred basal to land plants.

Vertebrates’ ortholog sequences were grouped in a clade consisting of nineteen mammalian species, nine birds, two reptiles, one amphibian, and four fishes (Figure 3). They were separated into two branches, one containing *Homo sapiens* Ataxin-2 orthologs, and the other orthologs of the Ataxin-2-like paralog (highlighted in two tones of gray in Figure 3 and Additional file 4). For all sequences from mammalian, reptile, amphibian and fishes, one member was found in each one of the two branches. All birds contain the Ataxin-2 but not the Ataxin-2-like ortholog. It is likely that vertebrates, except for birds, encode one copy of each one of the two Ataxin-2 paralogs.

Two clades were resolved for invertebrates, one grouping the sixteen insects and the other one the two nematodes. Sequences for the two fungal phyla were in a single clade, except for the two Sordariomycetes (*Magnaporthe oryzae* MGG 09170 and *Neurospora crassa* NCU05400) and one Basidiomycete (*Puccinia graminis* PGTG 01299) which appeared in a phylogenetically incongruent position (Figure 3 and Additional file 4). The sixteen protist species’ sequences were distributed in two groups. One group of ten protist proteins, defined a clade (apicomplexans, ciliates, kinetoplasts and diatoms), and the other six sequences were basal to plants (amoebozoa, choanoflagellates, amoeboflagellate, oomycetes) (Figure 3 and Additional file 4).

All plant sequences were grouped into two sister clades. One clade included the CID3/CID4 class and the other clade the CID16/CID17 class (highlighted in two tones of green in Figure 3 and Additional file 4). Sequences of all species analyzed were represented in both clades except for the two basal species, *Physcomitrella patens* and *Selaginella moellendorffii*, that are basal to the CID3/CID4 class. In these two species, the two putative Ataxin-2 orthologs that were identified belong to the CID3/CID4 class (Figure 3 and Additional file 4).

### Position specific probability matrix (PSPM) logos for visualization of the sequence diversity of ataxin-2 proteins

To obtain a comprehensive view of the domain architecture among Ataxin-2 proteins in different taxa, we conducted sequence motif (or logo) searches on the 216 Ataxin-2 amino acid sequences. The MEME suite was used to obtain the sequence logos (http://meme.nbcr.net/meme/), and the Interactive Tree Of Life (iTOL) software was helpful for generating the image of the protein domain architecture using shape and color codes (http://itol.embl.de/); a different color was used for each sequence logo. Logos were displayed together with the phylogenetic tree in Additional file 4 and the catalog of them in Additional file 5. In addition, the alignment of mammalian, insects and angiosperm proteins, locating the regions encompassing sequence logos are shown in Additional files 6, 7 and 8.

**Figure 4:**
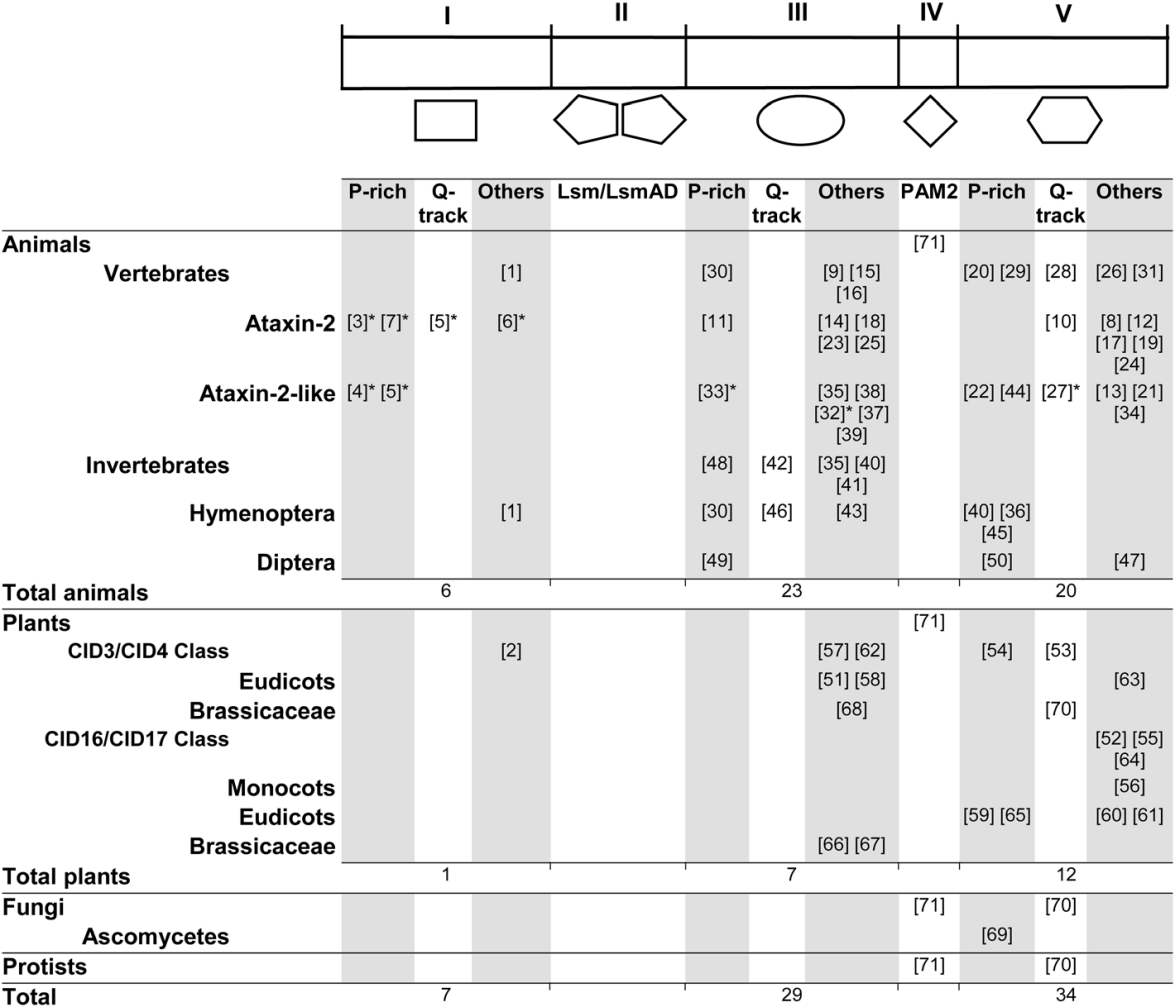
**Sequence logos mapped to Ataxin-2 modules.** Geometric figures represent the five modules and the sequence logos mapped to each region from: animals, plants, fungi and protists. The Lsm and LsmAD domains were not used in the search for logos, and a single logo was obtained for the PAM2 motif. The sum of logos in animals and plants is indicated, and the total number of logos is shown at the bottom. *logos specific for mammalian proteins. The catalog of the 71 sequence logos is displayed in Additional file 5.

Since Lsm and LsmAD are common domains to Ataxin-2 proteins, we directed our search to the regions flanking these domains: amino-terminus (region I, in Figure 1) and carboxy-terminus (regions III-IV-V, in Figure 1). Based on the PSPM sequence, seven non-redundant sequence logos were identified from the amino-terminus sequence and sixty-two from the carboxy-terminus sequence. A single PAM2 logo was generated for most Ataxin-2 proteins (logo 71). Logos confined to distinct groups were readily detected and mapped to Ataxin-2 regions; logos rich in proline residues (P-rich) or containing a polyglutamine tract (Q-tract), were also identified (see Figure 4 and Additional files 4 and 5). About 75% of all obtained logos corresponded to animal sequences, suggesting that Ataxin-2 proteins experienced diversification in animals. This augmented logo incidence may also be related to the gain of new motifs in animals. Conversely, only one logo was obtained from each fungi or protist protein, suggesting a high degree of sequence divergence among species from these two life domains.

Within the amino-terminus, few logos were expected since this is a smaller region than the carboxy-terminus, and even lacking in many of the orthologs (see Figure 1). One sequence logo was present in all animal sequences, except for insects of the Diptera order (logo 1), and five logos were specific for mammalian proteins: logos 3, 7 and 6 for Ataxin-2 proteins, logo 4 for Ataxin-2-like proteins, and logo 5 for all proteins. Most of these logos were of the proline-rich class. In plants, logo 2 was present in all CID3/CID4 proteins. These observations suggest that novelties at the amino-terminus were acquired in mammals.

A similar number of sequence logos were mapped to either region III or region V, indicating that these two regions may have a similar degree of sequence complexity. Likewise, there were logos present in all vertebrates’ sequences (logos 30, 9, 15, 16 20, 29, 28, 26, 31) and specific logos for Ataxin-2 or Ataxin-2-like proteins, some of which were specific to mammalian proteins (Figure 4 and Additional file 6). Hymenoptera and Diptera, the two better represented insect orders also displayed common and specific logos (Figure 4 and Additional file 7). These observations suggest that these groups display unique domain architecture. A meaningful fact is that several of the logos generated from animal proteins are of the P-rich class, suggesting that P-rich motifs are important for sequence diversity.

About 25% of the sequence logos generated on Ataxin-2 proteins corresponded to plants and all, except for logo 2, mapped to regions III and V (seven to region III and eleven to region V). Noteworthy, only three logos consisted of P-rich sequences (Figure 4). Distinct logos occurred for the two classes of Ataxin-2 proteins found in plants, CID3/CID4 and CID16/CID17. Moreover, in region III more logos were identified for the CID3/CID4 class and in region V, more were identified for the CID16/CID17class (Figure 4 and Additional file 8).

### The PAM2 motif across eukaryotic ataxin-2 proteins

**Figure 5:**
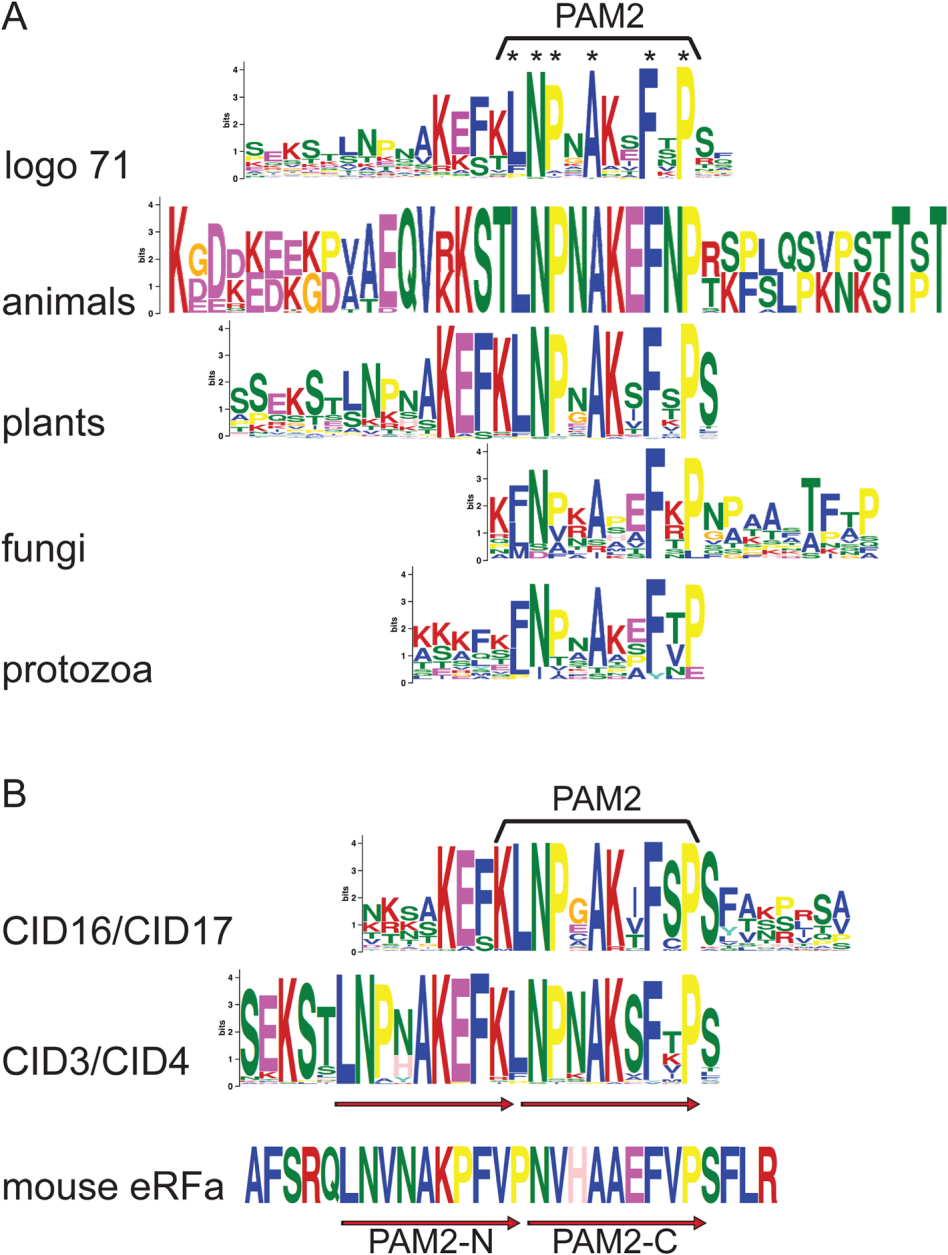
**Single and overlapping PAM2 motifs are present in Ataxin-2 proteins. (A)** Alignment of logo 71 and sequence logos containing PAM2 sequences generated independently from each one of the four domains: animals, plants, fungi and protists; asterisks point to conserved residues in all groups. **(B)** Alignment of sequence logos containing the PAM2 motif generated from the two classes of Ataxin-2 proteins identified in plants. Overlapping sequences are indicated by arrows; overlapping PAM2 motif of eRFa is shown as a reference.

The MLLE domain of PABP interacts directly with the PAM2 motif. PAM2 is an evolutionary conserved motif present in diverse proteins. To appraise the conservation of the PAM2 motif in Ataxin-2 proteins, we generated sequence logos independently from sequences from 56 animals, 40 plants, 15 fungi, and 16 protozoa. A single sequence logo that mapped to PAM2 related sequence was identified for each group. These PAM2 logos and logo 71 showed the same conserved residues in the motif (see Figure 5). In twelve of the 216 Ataxin-2 orthologs, a PAM2-related sequence logo was not obtained. Inspection of these twelve sequences revealed that in four of them the DNA sequence of the carboxi-terminus was inaccurate or missing (*Panicum virgatum* Pavirv00049508m, *Zea mays* GRMZM2G056773, *Citrus sinensis* orange1.1g045896m and *Phaseolus vulgaris* Phvul.010G133000.1) or the segment encompassing the PAM2 motif was deleted (*Thellungiella halophila* Thhalv10015348m, *Linum usitatissimum* Lus10037656 and *Tribolium castaneum* 660366). In two cases, the PAM2 motif may be absent or may have diverged, since the corresponding logo was not detected: in the two nematode (*Caenorhabditis elegans* NP001255079.1 and *Loa loa* LOAG10217) and three of the six Saccharomycetes analyzed (*Saccharomyces cerevisiae* YGR178C PBP1, *Kluyveromyces lactis* KLLA0F15158g and *Candida glabrata* CAGL0D05896g). This observation is in accordance with the fact that the PAM2 motif has not been identified in *S. cerevisiae* and that a distantly related *C. elegans* PAM2 motif was previously inferred; nevertheless, the yeast and the *C. elegans* orthologs interact with PABP [27,28]. A proline- and methionine-rich region preceding the MLLE domain in Pbp1p, the yeast ortholog, appears to mediate this interaction [29].

The sequence logo generation revealed conservation among PAM2 motifs across eukaryotes in an eleven residues region, on which six residues are predominant (enclosed in a rectangle and marked with an asterisk, Figure 5A). Distinctiveness among the flanking regions was also observed. The prevalence of charged amino acid residues flanking the motif towards the amino side in animals and protozoa was evident, as was the abundance of hydroxylic amino acids serine and threonine flanking to the carboxy side in animals. Plants showed two overlapping PAM2 motifs. This disposition was evident when logos were generated separately from the two classes, CID3/CID4 and CID16/CID17 (Figure 5B). The CID16/CID17 orthologs showed a single PAM2 motif whereas the CID3/CID4 class exhibited two overlapping motifs. Reiterated PAM2 motifs were previously identified in the mammalian eukaryotic releasing factor eRFa (Figure 5B) [10].

### Long polyglutamine (polyQ) tracts are mainly confined to primates and insects (hymenoptera and diptera orders)

**Figure 6:**
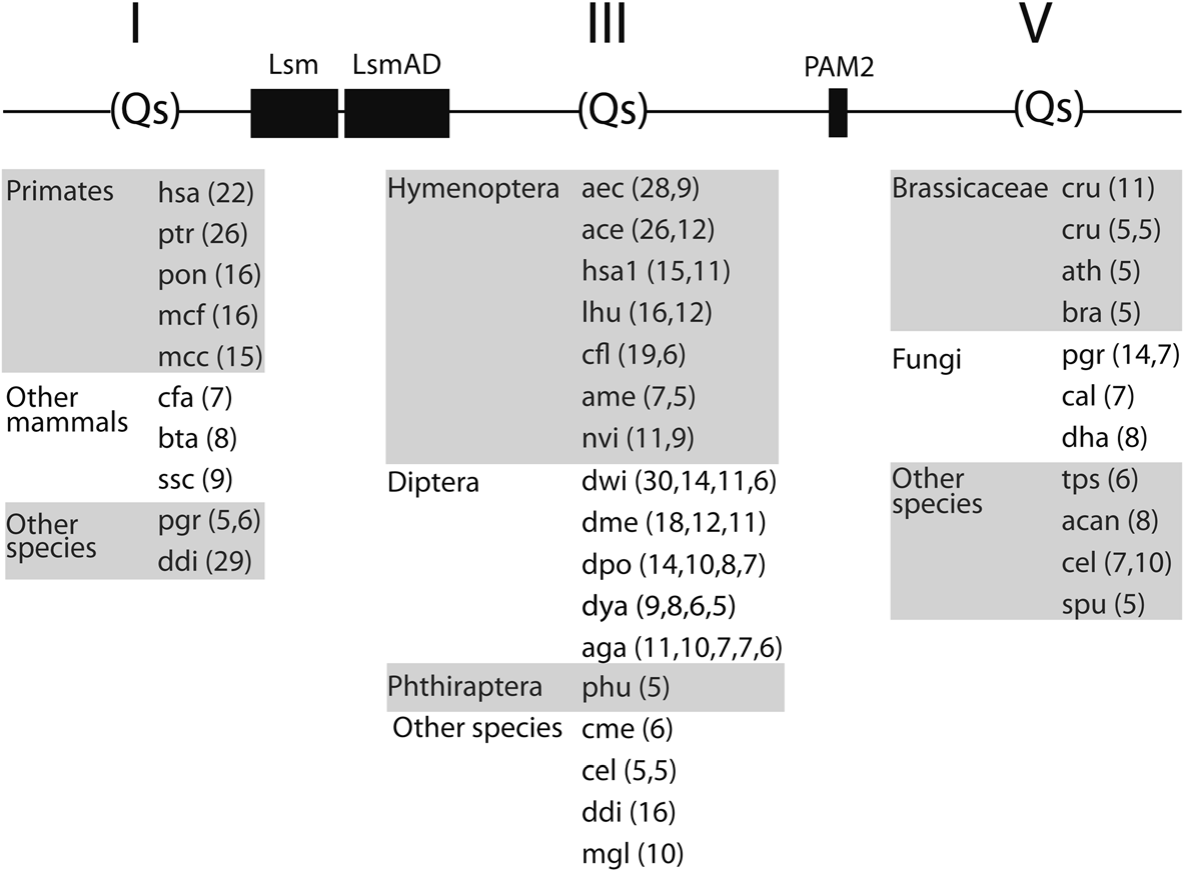
**Occurrence of polyQ tracts in Ataxin-2 proteins.** PolyQ tracts consisting of five of more glutamine residues were surveyed in the 216 retrieved Ataxin-2 proteins. Q-tract tracts are positioned within Ataxin-2 modules I, III or V. The number of glutamine residues in each tract is shown in parentheses; more than one Q-tract is separated by a comma. The species abbreviations are listed in Additional file 1.

A feature of the human Ataxin-2 protein is an uninterrupted polyQ tract of 22 repeats. It has been established that the expansion of the number of repeats over a particular limit is linked to disease. We surveyed the 216 putative Ataxin-2 orthologs for polyQ tracts of at least five uninterrupted glutamines. We detected long polyQ tracts in some lineages, and refer to them as long polyQ tracts expansions. More than one polyQ tract of different lengths can also occur in the same specie. We identified polyQ tracts near the amino-terminal region, between Lsm and PAM2 domains and to the carboxy-terminal region (see Figure 6). Like in the human Ataxin-2, the five primates analyzed contained polyQ tracts extending from 15 to 22 repeats, located at the amino-terminal region. Three mammals also showed polyQ tracts but with a minor number of repeats (Figure 6). This observation indicates that a polyQ tract expansion in the amino-terminal region of Ataxin-2 is restricted mainly to primates.

Among insects, all the species from the orders Hymenoptera and Diptera showed more that one polyQ tract. These tracts extend from 5 to 30 repeats and were exclusively located between the Lsm and PAM2 domains (Figure 6). Insects from 3 other orders (Lepidoptera, Coleoptera and Hemiptera), neither encoded polyQ tracts nor contained a single tract of 5 repeats (*Pediculus humanuds corporis* of the Phthiraptera order) (Figure 6).

Few polyQ tracts were identified in plants as well as in fungi and protists. In plants, they consisted of few repeats present only in three Brassicaceae species and in the red algae *Cyanidioschyzon merolae*. A single tract occurred in *Malassezia globosa*, *Candida albicans* and *Debaryomyces* hansenii and four tracts in *Puccinia graminis*. Among protists, *Thalassiosira pseudonana*, *Acanthamoeba castellanii* and *Dictyostelium discoideum* contained polyQ tracts, and notably in *D. discoideum* they were long repeats (Figure 6). In three distantly related species more than one polyQ tract was identified in different regions (the basidiomycete *Puccinia graminis*, the protist *Dictyostelium discoideum,* and the nematode *Caenorhabditis elegans*, in Figure 6). For instance, *C. elegans* contained two polyQ tracts between the Lsm and PAM2 domains and two at the carboxy-terminal region.

### Proline-rich motifs are common to ataxin-2 orthologs

**Figure 7:**
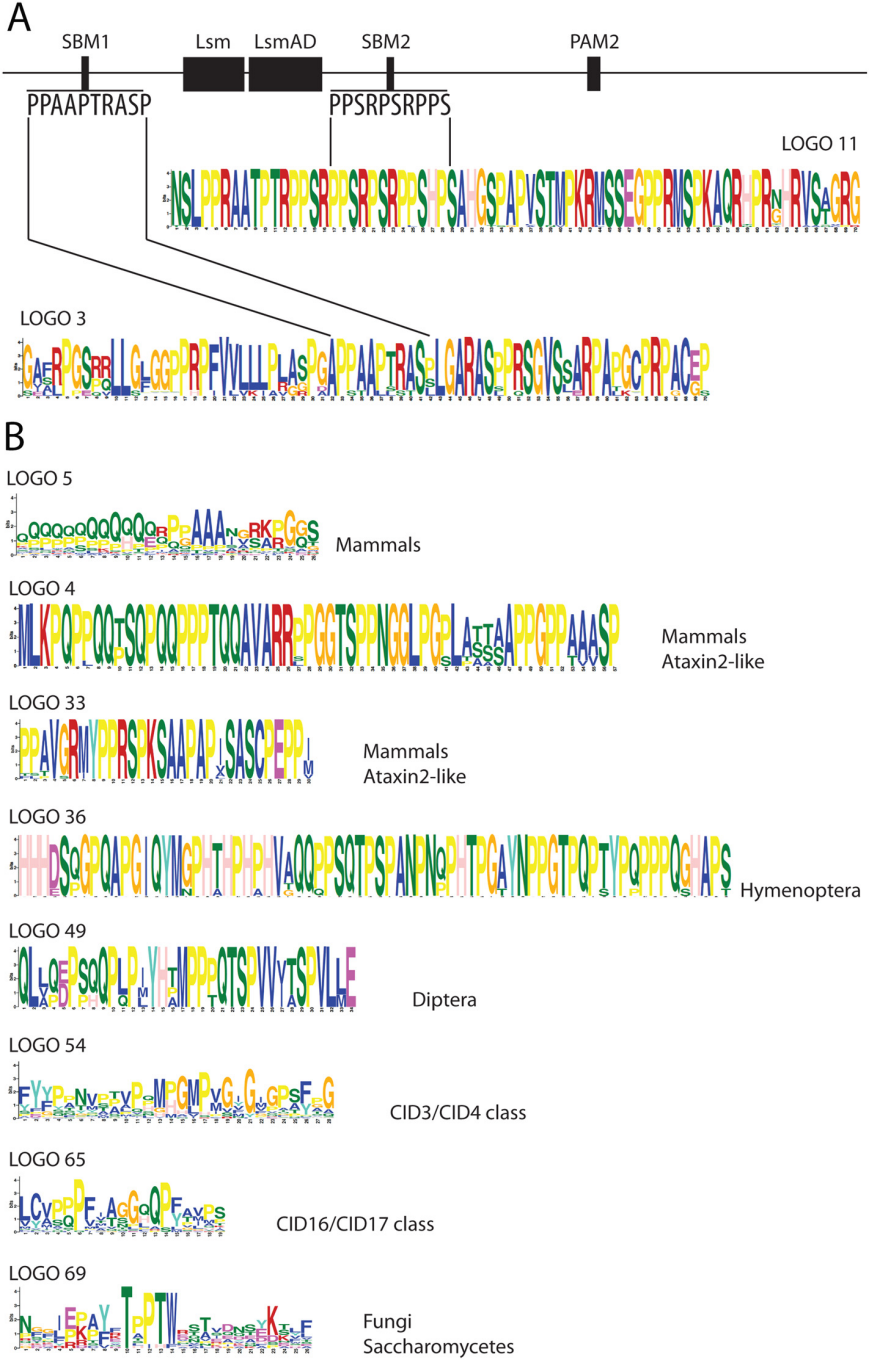
**Occurrence of proline-rich motifs in Ataxin-2 proteins. (A)** Alignment of SBM1 and SBM2 motifs to logo 3 and logo 11, respectively. **(B)** List of logos that contain proline-rich sequences, indicating the group of species on which they were generated. The entire catalog of sequence logos is shown in Additional file 5.

Previously work established that two proline-rich motifs, named SBM1 and SBM2, of the human Ataxin-2 protein interact with the SH3 domain within the endophilin proteins [30]. Since proline-rich motifs may be important for Ataxin-2 function, we were interested in defining whether such motif was common to other Ataxin-2 orthologs. From our sequence logo catalogue, we retrieved two logos that included the proline-rich motifs SBM1 and SBM2 (Figure 7A). The 10 amino acid residues SBM1 and SBM2 motifs were included within two 70 residues long logos (logos 3 and logo 12, respectively). These two logos were specifically generated in Ataxin-2 orthologs in mammals, suggesting that the interaction with endophilin is unique to mammalian Ataxin-2 proteins. Nevertheless, we cannot discard the fact that other P-rich sequences may also bind to the SH3 domain of endophilins.

Further inspection of the sequence logo catalogue revealed that approximately 30% of the logos contained P-rich sequences, and while a similar number was found in regions I, III and V, they were more frequent in animal proteins than in plants, fungi or protists (Figure 4). Among the amino-terminus logos, P-rich logos were specifically found in mammalian proteins, and they were present in both Ataxin-2 (logos 3, 7) and Ataxin-2-like proteins (logos 4, 5). Noteworthy, part of logo 5 consisted of a proline tract in Ataxin-2-like or a glutamine tract in the Ataxin-2 orthologs (Figure 4, see sequence in Figure 7B and alignment in Additional file 7). Since Q-tracts and P-rich regions are important for Ataxin-2 function, it is possible that these associated sequences might have been important for the diversification of its molecular function.

Specific P-rich logos can also be indentified in the mammalian Ataxin-2-like proteins (logo 4 and logo 33, Figure 7B), as well as in insects of the Hymenoptera and Diptera orders, or to the two classes of Ataxin-2 proteins found in plants (Figure 4, see sequences in Figure 7B and alignments in Additional files 6 and 7: Figures S6 and S7). These observations indicate that proline-rich motifs is a widespread trait in Ataxin-2 orthologs, but more prevalent in animal lineages that in plants.

## Conclusions

This study represents the first genome-wide survey and domain analysis of Ataxin-2 proteins across eukaryotes. With the exception of vertebrates and land plants, a single ortholog was identified in the 127 selected species. The two orthologs in vertebrates may have been generated during an early genome duplication event that renders two copies: Ataxin-2 and Ataxin-2-like. Interestingly, only the Ataxin-2 paralog was maintained in avian species; the outcome of this loss is unknown. Expansion up to six genes was observed in plants, and a new class of Ataxin-2 proteins that lacks the LsmAD domain was identified in angiosperms. Thus, functional specialization can be inferred for these Ataxin-2 orthologs. The absence of LsmAD in this new class points to a different subcellular distribution and functional properties, and the tandem reiteration of the PAM2 motif may provide with distinct functional properties to the Ataxin-2/CID3/CID4 class, as previously described for eRF3. A noteworthy observation is that the polyglutamine tracts may be a specialization that distinctively occurred in Ataxin-2 in primates. Our analysis also provides information regarding unexplored orthologs that may be useful to understand in detail the role of Ataxin-2 proteins in mRNA metabolism.

## Methods

### Sequence identification and retrieval

To retrieve sequences, we performed BLAST searches using the human and the *Arabidopsis thaliana* CID3 Lsm domains as queries (73 and 77 amino acid residues long, respectively). Sequences with an expect threshold lower than 2e^−20^ that were part of a full length or almost full length Ataxin-2 peptide were regarded as orthologs. Small fragments or truncated peptides that after inspection of adjoining sequences could not be readily assembled into a full length or almost full length peptide, were not accepted. The peptide sequences used in this study from animal, fungus, and protist genomes were retrieved from the Kyoto Encyclopedia of Genes and Genomes (KEGG) at http://www.genome.jp/kegg/ and the insect sequence from the Hymenoptera order from the Ant Genomes Portal at http://hymenopteragenome.org/ant_genomes[31]; they included 3 basal animals, 19 mammals, 9 birds, 2 reptiles, 1 amphibian, 4 fishes, 16 insects, 2 nematodes, 15 fungi, and 16 protists. The viridiplantae peptide sequences were retrieved from the genomes deposited in the Phytozome v9.1 database at http://www.phytozome.net/. They included 6 chlorophyte, 2 basal plants, 6 monocots, and 26 eudicot plants (species and genes are listed in Additional file 1). Three truncated sequences were readily assembled into Ataxin-2 genes by visual inspection, *Manihot esculenta* cassava4.1 014104 m, *Solanum tuberosum* PGSC0003DMP400008702 and *Sorghum bicolor* v1.4 Sb04g019250.1.

### Phylogenetic analyses and sequence alignments

Ataxin-2 phylogenetic trees were derived from either complete protein sequences or on the Lsm domain; the 216 retrieved Ataxin-2 sequences were aligned using ClustalX 2.0.12 [32]. In the phylogenetic analysis, Maximum-likelihood (ML), Neighbor-joining (NJ) and Maximun-parsimony (MP) trees were obtained using MEGA 5 [33]. In NJ and MP phylogenetic analysis, 1000 bootstrap replicates were obtained. For ML, the JTT model was used with 20 gamma categories (Gamma20) and the posterior probabilities support values for each node was computed by resampling 1,000 times [34]. We have previously tested similar models in the analysis of other gene families [26,35]. The phylogenies for Ataxin-2 sequences obtained with NJ, ML, and MP, were assessed to compare their agree with conventional taxonomic classification in Order, Family and Genera [36]. The trees generated with the Lsm domain showed better resolution of species and more strongly supported branches (data not shown). The tree phylogeny was displayed and edited by iTOL (Interactive Tree Of Life) at http://itol.embl.de/[37]. We opted for a color code as described in Figure 2.

### Generation of sequence logos

Conserved motifs in Ataxin-2 proteins were searched using Multiple EM for Motif Elicitation (MEME) version 4.9.1 at http://meme.nbcr.net/meme/cgi-bin/meme.cgi[38]; the following parameters were used: zero or one per sequence, 10 and 135 amino acids as minimum and maximum sizes of motifs; the e-value cutoff was less than e-10. Searched did not included the Lsm and LsmAD sequences. Sequence logos containing PAM2 sequences were readily identified, other generated logos were mapped to the Ataxin-2 modules. To visualize simultaneously the phylogeny and the predicted MEME conserved motifs, we represented each Ataxin-2 region with one shape symbol as follows: I, square; III, ellipse; IV, rhombus; V, horizontal hexagon; coloring with several different colors if there were more than one conserved motif in an Ataxin-2 region. Logos were not searched from region II, pentagon shapes were used for this region

## Abbreviations

SCA2: Spinocerebellar ataxia type 2; Lsm: Like RNA splicing domain Sm1 and Sm2; LsmAD: Like-Sm-associated domain; PAM2: Poly (A)-binding protein interacting motif 2; MLLE: MademoiseLLE; P-rich: Proline rich motif; Q-tract: Polyglutamine tracts.

## Competing interests

The authors declare that they have no competing interests.

## Authors’ contributions

DJ-L carried out the database searches and the bioinformatic analyses. DJ-l and PG conceived this study and analyzed the data. PG wrote the manuscript. Both authors have read and approved the final version of the manuscript.

## Supplementary Material

Additional file 1 List of retrieved Ataxin-2 genes from animals, fungi, protists and plants. Click here for file

Additional file 2 **Protein sequence alignment of the CID3, CID4, CID16 and CID17.** The sequence alignments were performed using ClustalX 2.0.12; default colors were used. Regions encompassing sequence Lsm, LsmAD and PAM2 motifs are enclosed by rectangles. Click here for file

Additional file 3 **Phylogenetic trees of Ataxin-2 proteins based on Lsm domain.** The topologies were generated by the (A) ML and (B) MP methods; statistical significance in and MP, and posterior probability above 0.5 for ML methods is indicated on the nodes. Species and gene names are as mentioned in Additional file 1. Color codes of branches are as depicted in Figure 2. Vertebrate proteins are shadowed in gray tones (Ataxin-2, in dark gray and Ataxin-2-like in light gray) and plant proteins in green tones (CID3/CID4 class in dark green and CID16/CID17 class in light green). Click here for file

Additional file 4 **Phylogenetic tree of Ataxin-2 proteins based on Lsm domain.** The topology were generated by the NJ method; statistical significance in percentages above 50% for NJ. The domain architecture based on sequence logos is depicted next to the phylogenetic tree. Click here for file

Additional file 5 Catalog of 71 sequence logos generated from 216 Ataxin-2 proteins. Click here for file

Additional file 6 **Alignment of the Ataxin-2 proteins from mammals.** ClustalX 2.0.12 was used for sequence alignment and a default color code was applied. The location of regions encompassing sequence logos are enclosed by red rectangles and the Lsm, LsmAD and PAM2 domains by black rectangles. Click here for file

Additional file 7 **Alignment of the Ataxin-2 proteins from insects (Orders Hymenoptera and Diptera).** The sequence alignments were performed as described in Additional file 6. Click here for file

Additional file 8 **Alignment of the Ataxin-2 proteins from angiosperms.** The sequence alignments were performed as described in Additional file 6. Click here for file
