# Supplementary material for: Insights into the evolution and domain structure of ataxin-2 proteins across eukaryotes
Source: BMC Res Notes. 2014 Jul 15;7:453. doi: 10.1186/1756-0500-7-453 (PMC4105795; doi:10.1186/1756-0500-7-453)
Supplement: Additional file 1 — List of retrieved Ataxin-2 genes from animals, fungi, protists and plants. [file 1756-0500-7-453-S1.pdf]

	Code	Species	Genes
Primates	hsa	*Homo sapiens*	**6311_ATXN2**
			**11273_ATXN2L**
	ptr	*Pan troglodytes*	**452247**
			**454017**
	pon	*Pongo abelii*	**100433569**
			**100454124**
	mcc	*Macaca mulatta*	**710742**
			**708359**
	mcf	*Macaca fascicularis*	**101926470**
			**102117238**
	mmu	*Mus musculus*	**20239**
			**233871**
Other mammals	rno	*Rattus norvegicus*	**288663**
			**361649**
	cge	*Cricetulus griseus*	**100772811**
			**100765372**
	hgl	*Heterocephalus glaber*	**101726106**
			**101717578**
	cfa	*Canis familiaris*	**486270**
			**489951**
	bta	*Bos taurus*	**532649**
			**539507**
	bom	*Bos mutus*	**102279571**
			**102276261**
	phd	*Pantholops hodgsonii*	**102343138**
			**102323591**
	chx	*Capra hircus*	**102181918**
			**102180146**
	ssc	*Sus scrofa*	**100153708**
			**100515946**
	ecb	*Equus caballus*	**100057794**
			**100066373**
	myb	*Myotis brandtii*	**102257957**
			**102260634**
	mdo	*Monodelphis domestica*	**100017952**
			**100014802**
	shr	*Sarcophilus harrisii*	**100919478**
			**100918446**
Birds	gga	*Gallus gallus*	**416877**
	mgp	*Meleagris gallopavo*	**100548051**
	tgu	*Taeniopygia guttata*	**100220881**
	fab	*Ficedula albicollis*	**101812918**
	phi	*Pseudopodoces humilis*	**102102442**
	apla	*Anas platyrhynchos*	**101803795**
	fpg	*Falco peregrinus*	**101924450**
	fch	*Falco cherrug*	**102046876**
	clv	*Columba livia*	**102085007**
Reptiles	acs	*Anolis carolinensis*	**100551565**
			**100564603**
	asn	*Alligator sinensis*	**102374524**
			**102377706**
Amphibians	xtr	*Xenopus tropicalis*	**100490147**
			**100216248**

Fishes	dre	*Danio rerio*	**569210**
			**324525**
	tru	*Takifugu rubripes*	**101062795**
			**101069808**
	ola	*Oryzias latipes*	**101175201**
			**101160663**
	lcm	*Latimeria chalumnae*	**102361169**
			**102360058**
Echinoderms (Basal Animal)	spu	*Strongylocentrotus purpuratus*	**581517**
Diptera (Insects)	dme	*Drosophila melanogaster*	**Dmel_CG5166**
	dpo	*Drosophila pseudoobscura pseudoobscura*	**Dpse_GA18704**
	dwi	*Drosophila willistoni*	**Dwil_GK11664**
	dya	*Drosophila yakuba*	**Dyak_GE24310**
	aga	*Anopheles gambiae*	**AgaP_AGAP013455**
Other insects	tca	*Tribolium castaneum*	**660366**
	bmor	*Bombyx mori*	**101743989**
	api	*Acyrthosiphon pisum*	**100163910**
	phu	*Pediculus humanus corporis*	**Phum_PHUM213790**
Hymenoptera (Insects)	ame	*Apis mellifera*	**409151**
	nvi	*Nasonia vitripennis*	**100120335**
	aec	*Acromyrmex echinatior*	**AECH22559-PA**
	ace	*Atta cephalotes*	**ACEP16121-PA**
	cfl	*Camponotus floridanus*	**CFLO18886-PA**
	hsal	*Harpegnathos saltator*	**HSAL22900-PA**
	lhu	*Linepithema humile*	**LH10746-PA**
Nemmatodes	cel	*Caenorhabditis elegans*	**NP_001255079.1**
	loa	*Loa loa*	**LOAG_10217**
Cnidarians (Basal Animal)	nve	*Nematostella vectensis*	**NEMVE_v1g243962**
Placozoans (Basal Animal)	tad	*Trichoplax adhaerens*	**TRIADDRAFT_51569**
Eudicots	mes	*Manihot esculenta*	**cassava4.1_003097m**
			**cassava4.1_003544m**
			**cassava4.1_004220m**
			**cassava4.1_014104m.g**
	rco	*Ricinus communis*	**29706.m001306**
			**29666.m001432**
	lus	*Linum usitatissimum*	**Lus10037656**
			**Lus10015638**
			**Lus10040755**
			**Lus10016477**
	pop	*Populus trichocarpa*	**Potri.003G063000.1**
			**Potri.001G171100.1**
			**Potri.011G138600.1**
			**Potri.001G423200.1**
	mtr	*Medicago truncatula*	**Medtr2g099480.1**
			**Medtr3g051270.1**
	pvu	*Phaseolus vulgaris*	**Phvul.005G156000.1**
			**Phvul.010G133000.1**
			**Phvul.006G042100.1**
	gmx	*Glycine max*	**Glyma15g03190.1**
			**Glyma13g42230.4**
			**Glyma16g00765.1**
			**Glyma07g04060.5**
			**Glyma08g42700.4**
			**Glyma18g11551.1**
	csa	*Cucumis sativus*	**Cucsa.340860.1**
			**Cucsa.059890.1**
	ppe	*Prunus persica*	**ppa002829m**
			**ppa004888m**
	fve	*Fragaria vesca*	**mrna07523.1-v1.0-hybrid**

			**mrna03364.1-v1.0-hybrid**
	ath	*Arabidopsis thaliana*	**At1g54170.1_CID3**
			**At3g14010.1_CID4**
			**AT4G26990.1_CID16**
			**AT5G54920.2_CID17**
	aly	*Arabidopsis lyrata*	**474549**
			**929172**
			**492097**
			**495575**
	cru	*Capsella rubella*	**Carubv10008656m**
			**Carubv10013292m**
			**Carubv10007224m**
			**Carubv10026219m**
	bra	*Brassica rapa*	**Bra037969**
			**Bra014367**
			**Bra021526**
			**Bra001533**
			**Bra026388**
			**Bra019079**
	tha	*Thellungiella halophila*	**Thhalv10011331m**
			**Thhalv10020519m**
			**Thhalv10015348m**
			**Thhalv10025144m**
	cpa	*Carica papaya*	**evm.model.supercontig_25.97**
			**evm.model.supercontig_97.11**
	gra	*Gossypium raimondii*	**Gorai.010G234100.1**
			**Gorai.012G011100.1**
			**Gorai.009G437800.1**
	tco	*Theobroma cacao*	**Thecc1EG029104t3**
			**Thecc1EG030903t1**
	csi	*Citrus sinensis*	**orange1.1g045896m**
			**orange1.1g038314m**
	ccl	*Citrus clementina*	**Ciclev10025086m**
			**Ciclev10007877m**
	egr	*Eucalyptus grandis*	**Eucgr.F03170.1**
			**Eucgr.F00770.1**
	vvi	*Vitis vinifera*	**GSVIVT01016786001**
			**GSVIVT01037637001**
	stu	*Solanum tuberosum*	**PGSC0003DMP400008702**
			**PGSC0003DMP400038691**
	sly	*Solanum lycopersicum*	**Solyc06g062860.2.1**
			**Solyc03g121950.2.1**
			**Solyc07g064550.2.1**
	mgu	*Mimulus guttatus v2.0*	**Migut.D00945.1.p**
			**Migut.M01771.1.p**
	aco	*Aquilegia coerulea*	**Aquca_076_00117.1**
			**Aquca_011_00036.1**
Monocots	sbi	*Sorgum bicolor v1.4*	**Sb04g019250.1**
			**Sb06g029190.1**
	zma	*Zea mays*	**GRMZM5G829738_T01**
			**GRMZM2G056773_T01**
			**GRMZM2G012088_T01**
			**GRMZM2G150901_T03**
	sit	*Setaria italica*	**Si016687m**
			**Si010063m**
	pvi	*Panicum virgatum*	**Pavirv00049508m**
			**PPavirv00038354m**
			**Pavirv00018461m**
			**Pavirv00019389m**

	osa	*Oryza sativa*	**LOC_Os02g27950.1**
			**LOC_Os04g53450.1**
	bdi	*Brachypodium distachyon*	**Bradi1g56090.1**
			**Bradi5g22420.1**
Ferns (Basal Plant)	smo	*Selaginella moellendorffii*	**411870**
			**437867**
Mosses (Basal Plant)	ppp	*Physcomitrella patens v1.6*	**Pp1s358_21V6.1**
			**Pp1s59_306V6.1**
Green algae	cre	*Chlamydomonas reinhardtii*	**g1267.t1**
	vcn	*Volvox carteri*	**Vocar20009639m**
	mpp	*Micromonas pusilla CCMP1545*	**211045**
	mis	*Micromonas sp. RCC299*	**64598**
	olu	*Ostreococcus lucimarinus*	**OSTLU_24476**
Red algae	cme	*Cyanidioschyzon merolae*	**CMK070C**
Ascomycetes	sce	*Saccharomyces cerevisiae*	**YGR178C_PBP1**
	kla	*Kluyveromyces lactis*	**KLLA0F15158g**
	cgr	*Candida glabrata*	**CAGL0D05896g**
	dha	*Debaryomyces hansenii*	**DEHA2G03652g**
	cal	*Candida albicans*	**CaO19.12914**
	yli	*Yarrowia lipolytica*	**YALI0B23232g**
	ncr	*Neurospora crassa*	**NCU05400**
	mgr	*Magnaporthe oryzae*	**MGG_09170**
	ang	*Aspergillus niger*	**ANI_1_186074**
	cim	*Coccidioides immitis*	**CIMG_07100**
	pno	*Phaeosphaeria nodorum*	**SNOG_0526**
	tml	*Tuber melanosporum*	**GSTUM_00011755001**
Basidiomycetes	uma	*Ustilago maydis*	**UM02637.1**
	mgl	*Malassezia globosa*	**MGL_1239**
	pgr	*Puccinia graminis*	**PGTG_01299**
Choanoflagellates (Protist)	mbr	*Monosiga brevicollis*	**MONBRDRAFT_28426**
Amoeboflagellate (Protist)	ngr	*Naegleria gruberi*	**NAEGRDRAFT_78066**
Amoebozoa (Protists)	ddi	*Dictyostelium discoideum*	**DDB_G0269682**
	dpp	*Dictyostelium purpureum*	**DICPUDRAFT_98676**
	acan	*Acanthamoeba castellanii*	**ACA1_058350**
Alveolates (Protists)	pfa	*Plasmodium falciparum 3D7*	**PF14_0338**
	pvx	*Plasmodium vivax*	**PVX_084750**
	tpv	*Theileria parva*	**TP04_0787**
	cho	*Cryptosporidium hominis*	**Chro.70349**
	tgo	*Toxoplasma gondii*	**TGME49_031440**
	ptm	*Paramecium tetraurelia*	**GSPATT00037771001**
Euglenozoa (Protists)	tbr	*Trypanosoma brucei*	**Tb927.8.4540**
	lbz	*Leishmania braziliensis*	**LBRM_10_1220**
Diatoms (Protists)	pti	*Phaeodactylum tricornutum*	**PHATRDRAFT_44892**
	tps	*Thalassiosira pseudonana*	**THAPS_23386**
Oomycetes (Protist)	pif	*Phytophthora infestans*	**PITG_01358**

Additional file 1. List of retrieved Ataxin-2 genes from animals, fungi, protists and plants.
