# Supplementary material for: Insights into the evolution and domain structure of ataxin-2 proteins across eukaryotes
Source: BMC Res Notes. 2014 Jul 15;7:453. doi: 10.1186/1756-0500-7-453 (PMC4105795; doi:10.1186/1756-0500-7-453)
Supplement: Additional file 3 — Phylogenetic trees of Ataxin-2 proteins based on Lsm domain. The topologies were generated by the (A) ML and (B) MP methods; statistical significance in and MP, and posterior probability above 0.5 for ML methods is indicated on the nodes. Species and gene names are as mentioned in Additional file 1. Color codes of branches are as depicted in Figure 2. Vertebrate proteins are shadowed in gray tones (Ataxin-2, in dark gray and Ataxin-2-like in light gray) and plant proteins in green tones (CID3/CID4 class in dark green and CID16/CID17 class in light green). [file 1756-0500-7-453-S3.pdf]

(A)

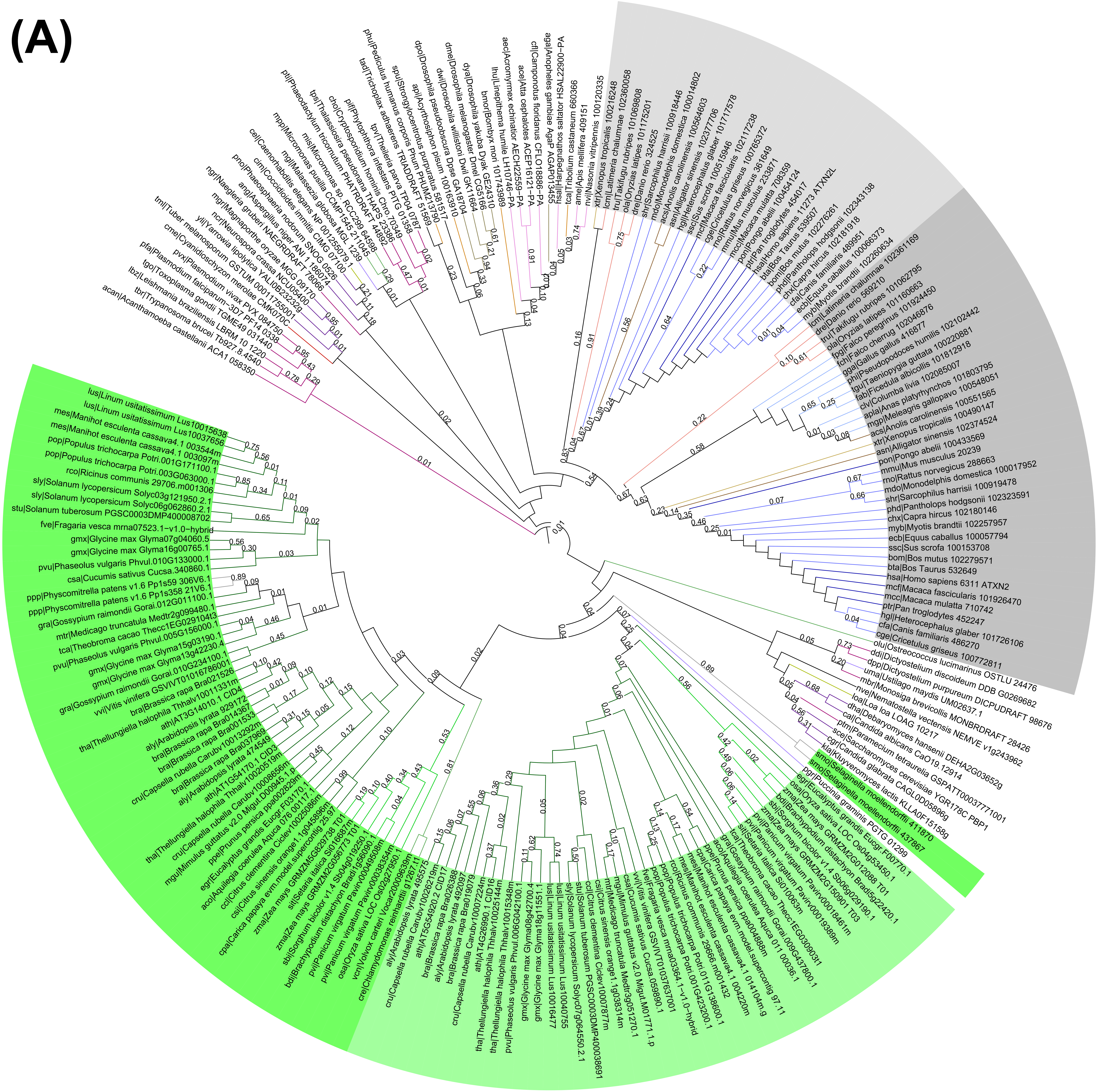

(B)

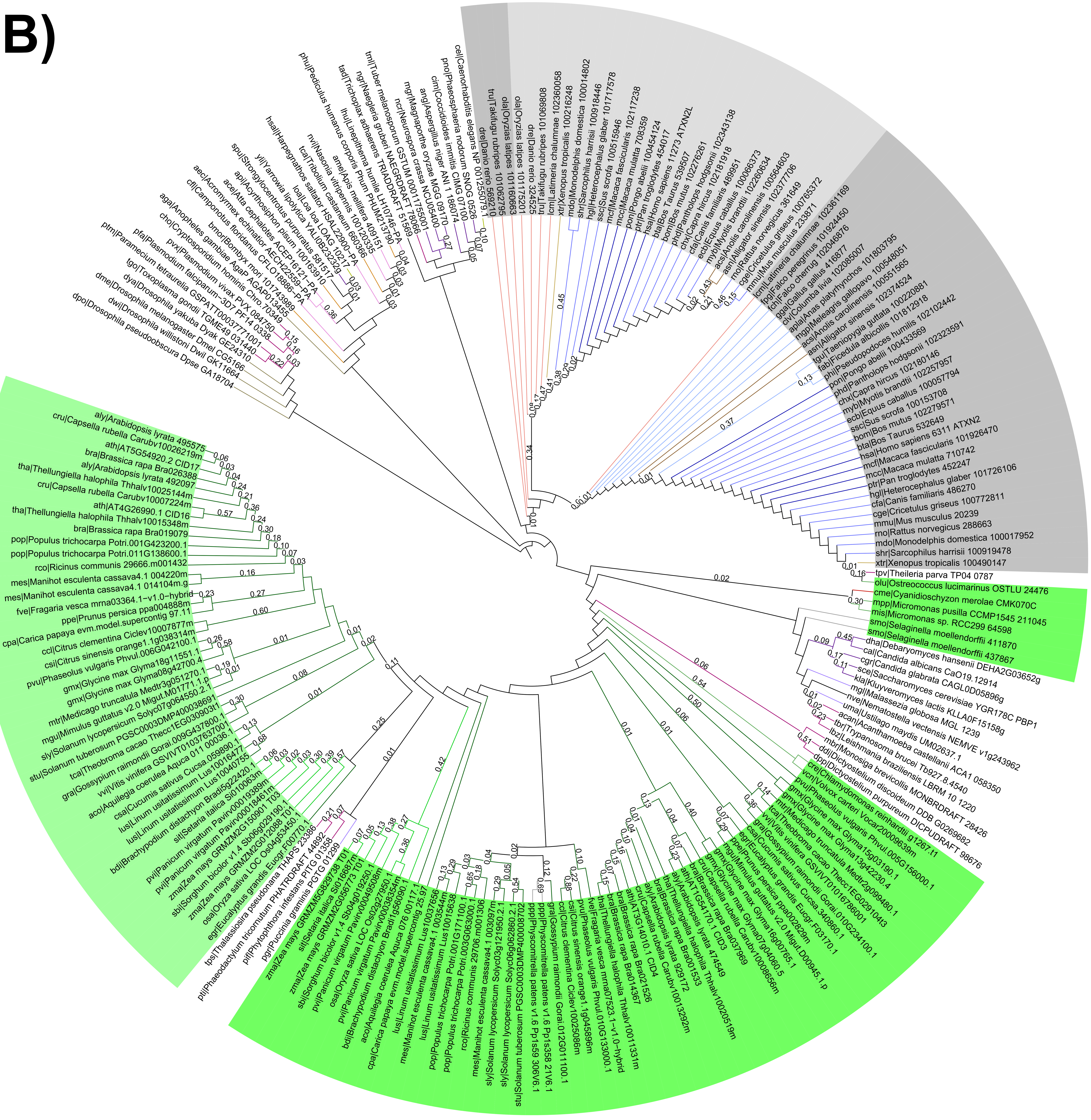

Additional file 3. Phylogenetic trees of Ataxin-2 proteins based on Lsm domain. The topologies were generated by the (A) ML and (B) MP methods; statistical significance in and MP, and posterior probability above 0.5 for ML methods is indicated on the nodes. Species and gene names are as mentioned in Additional file 1. Color codes of branches are as depicted in Figure 2. Vertebrate proteins are shadowed in gray tones (Ataxin-2, in dark gray and Ataxin-2-like in light gray) and plant proteins in green tones (CID3/CID4 class in dark green and CID16/CID17 class in light green).
