# Supplementary material for: Insights into the evolution and domain structure of ataxin-2 proteins across eukaryotes
Source: BMC Res Notes. 2014 Jul 15;7:453. doi: 10.1186/1756-0500-7-453 (PMC4105795; doi:10.1186/1756-0500-7-453)
Supplement: Additional file 4 — Phylogenetic tree of Ataxin-2 proteins based on Lsm domain. The topology were generated by the NJ method; statistical significance in percentages above 50% for NJ. The domain architecture based on sequence logos is depicted next to the phylogenetic tree. [file 1756-0500-7-453-S4.pdf]

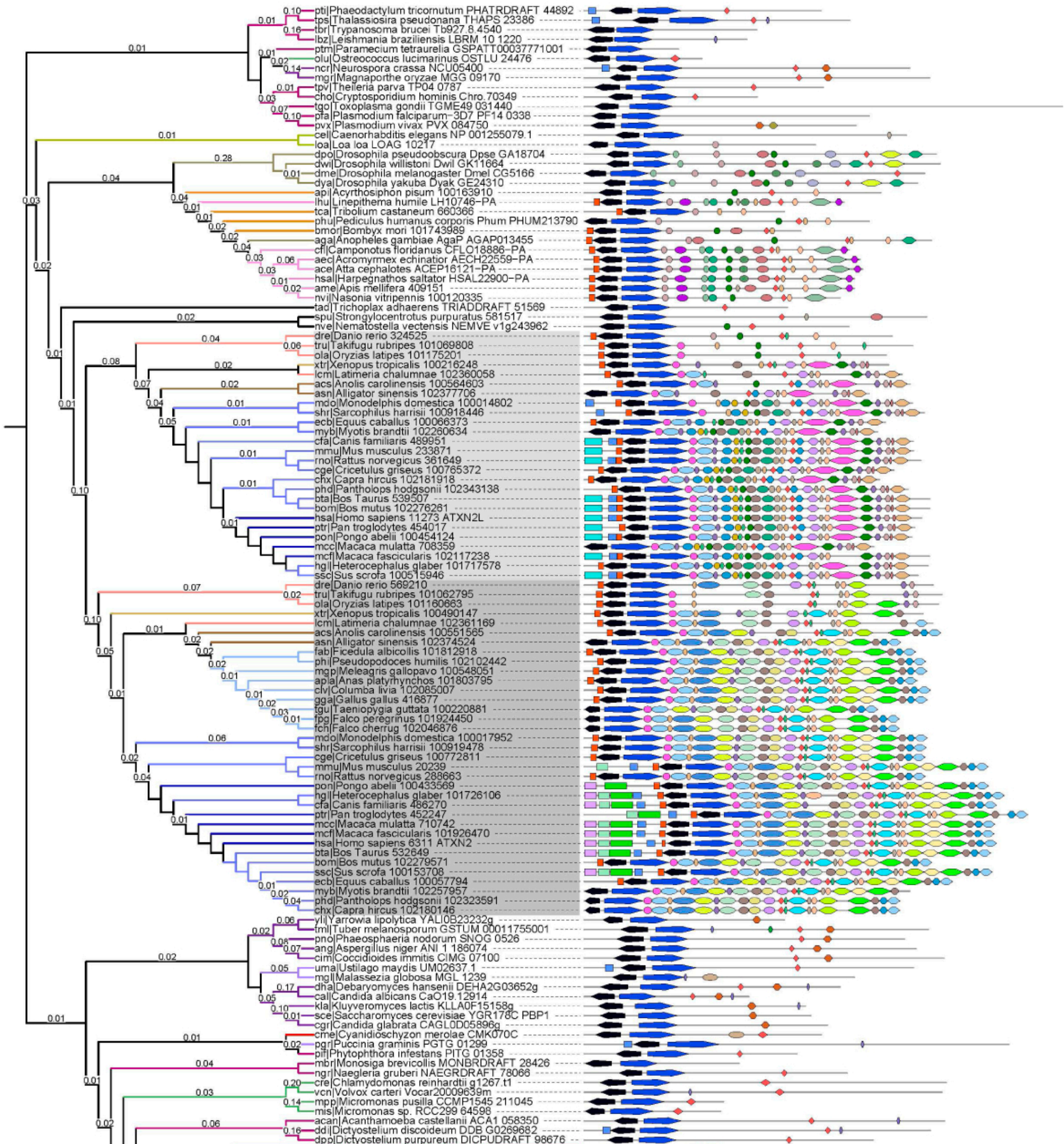
pti|Phaeodactylum tricornutum PHATRDRAFT 44892
tps|Thalassiosira pseudonana THAPS 23386
tbr|Trypanosoma brucei Tb927.8.4540
lbz|Leishmania braziliensis LBRM 10 1220
ptm|Paramecium tetraurelia GSPATT00037771001
olu|Ostreococcus lucimarinus OSTLU 24476
ncr|Neurospora crassa NCU05400
mgr|Magnaporthe oryzae MGG 09170
tpv|Theileria parva TP04 0787
cho|Cryptosporidium hominis Chro.70349
tgo|Toxoplasma gondii TGME49 031440
pfa|Plasmodium falciparum-3D7 PF14 0338
pvx|Plasmodium vivax PVX 084750
cel|Caenorhabditis elegans NP 001255079.1
loa|Loa loa LOAG 10217
dpo|Drosophila pseudoobscura Dpse GA18704
dwi|Drosophila willistoni Dwil GK11664
dme|Drosophila melanogaster Dmel CG5166
dya|Drosophila yakuba Dyak GE24310
api|Acyrthosiphon pisum 100163910
lhu|Linepithema humile LH10746-PA
tca|Tribolium castaneum 660366
phu|Pediculus humanus corporis Phum PHUM213790
bmor|Bombyx mori 101743989
aga|Anopheles gambiae AgaP AGAP013455
cfl|Camponotus floridanus CFLO18886-PA
aec|Acromyrmex echinatior AECH22559-PA
ace|Atta cephalotes ACEP16121-PA
hsal|Harpegnathos saltator HSAL22900-PA
ame|Apis mellifera 409151
nvi|Nasonia vitripennis 100120335
tad|Trichoplax adhaerens TRIADDRAFT 51569
spu|Strongylocentrotus purpuratus 581517
nve|Nematostella vectensis NEMVE v1g243962
dre|Danio rerio 324525
tru|Takifugu rubripes 101069808
ola|Oryzias latipes 101175201
xtr|Xenopus tropicalis 100216248
lcm|Latimeria chalumnae 102360058
acs|Anolis carolinensis 100564603
asn|Alligator sinensis 102377706
mdo|Monodelphis domestica 100014802
shr|Sarcophilus harrisii 100918446
ecb|Equus caballus 100066373
myb|Myotis brandtii 102260634
cfa|Canis familiaris 489951
mmu|Mus musculus 233871
rno|Rattus norvegicus 361649
cge|Cricetulus griseus 100765372
chx|Capra hircus 102181918
phd|Pantholops hodgsonii 102343138
bta|Bos Taurus 539507
bom|Bos mutus 102276261
hsa|Homo sapiens 11273 ATXN2L
ptr|Pan troglodytes 454017
pon|Pongo abelii 100454124
mcc|Macaca mulatta 708359
mcf|Macaca fascicularis 102117238
hgl|Heterocephalus glaber 101717578
ssc|Sus scrofa 100515946
dre|Danio rerio 569210
tru|Takifugu rubripes 101062795
ola|Oryzias latipes 101160663
xtr|Xenopus tropicalis 100490147
lcm|Latimeria chalumnae 102361169
acs|Anolis carolinensis 100551565
asn|Alligator sinensis 102374524
fab|Ficedula albicollis 101812918
phi|Pseudopodoces humilis 102102442
mgp|Meleagris gallopavo 100548051
apla|Anas platyrhynchos 101803795
clv|Columba livia 102085007
gga|Gallus gallus 416877
tgu|Taeniopygia guttata 100220881
fpg|Falco peregrinus 101924450
fch|Falco cherrug 102046876
mdo|Monodelphis domestica 100017952
shr|Sarcophilus harrisii 100919478
cge|Cricetulus griseus 100772811
mmu|Mus musculus 20239
rno|Rattus norvegicus 288663
pon|Pongo abelii 100433569
hgl|Heterocephalus glaber 101726106
cfa|Canis familiaris 486270
ptr|Pan troglodytes 452247
mcc|Macaca mulatta 710742
mcf|Macaca fascicularis 101926470
hsa|Homo sapiens 6311 ATXN2
bta|Bos Taurus 532649
bom|Bos mutus 102279571
ssc|Sus scrofa 100153708
ecb|Equus caballus 100057794
myb|Myotis brandtii 102257957
phd|Pantholops hodgsonii 102323591
chx|Capra hircus 102180146
yli|Yarrowia lipolytica YALI0B23232g
tml|Tuber melanosporum GSTUM 00011755001
pno|Phaeosphaeria nodorum SNOG 0526
ang|Aspergillus niger ANI 1 186074
cim|Coccidioides immitis CIMG 07100
uma|Ustilago maydis UM02637.1
mgl|Malassezia globosa MGL 1239
dha|Debaryomyces hansenii DEHA2G03652g
cal|Candida albicans CaO19.12914
kla|Kluyveromyces lactis KLLA0F15158g
sce|Saccharomyces cerevisiae YGR178C PBP1
cgr|Candida glabrata CAGL0D05896g
cme|Cyanidioschyzon merolae CMK070C
pgr|Puccinia graminis PGTG 01299
pif|Phytophthora infestans PITG 01358
mbr|Monosiga brevicollis MONBRDRAFT 28426
ngr|Naegleria gruberi NAEGRDRAFT 78066
cre|Chlamydomonas reinhardtii g1267.t1
vcn|Volvox carteri Vocar20009639m
mpp|Micromonas pusilla CCMP1545 211045
mis|Micromonas sp. RCC299 64598
acan|Acanthamoeba castellanii ACA1 058350
ddi|Dictyostelium discoideum DDB G0269682
dpp|Dictyostelium purpureum DICPUDRAFT 98676

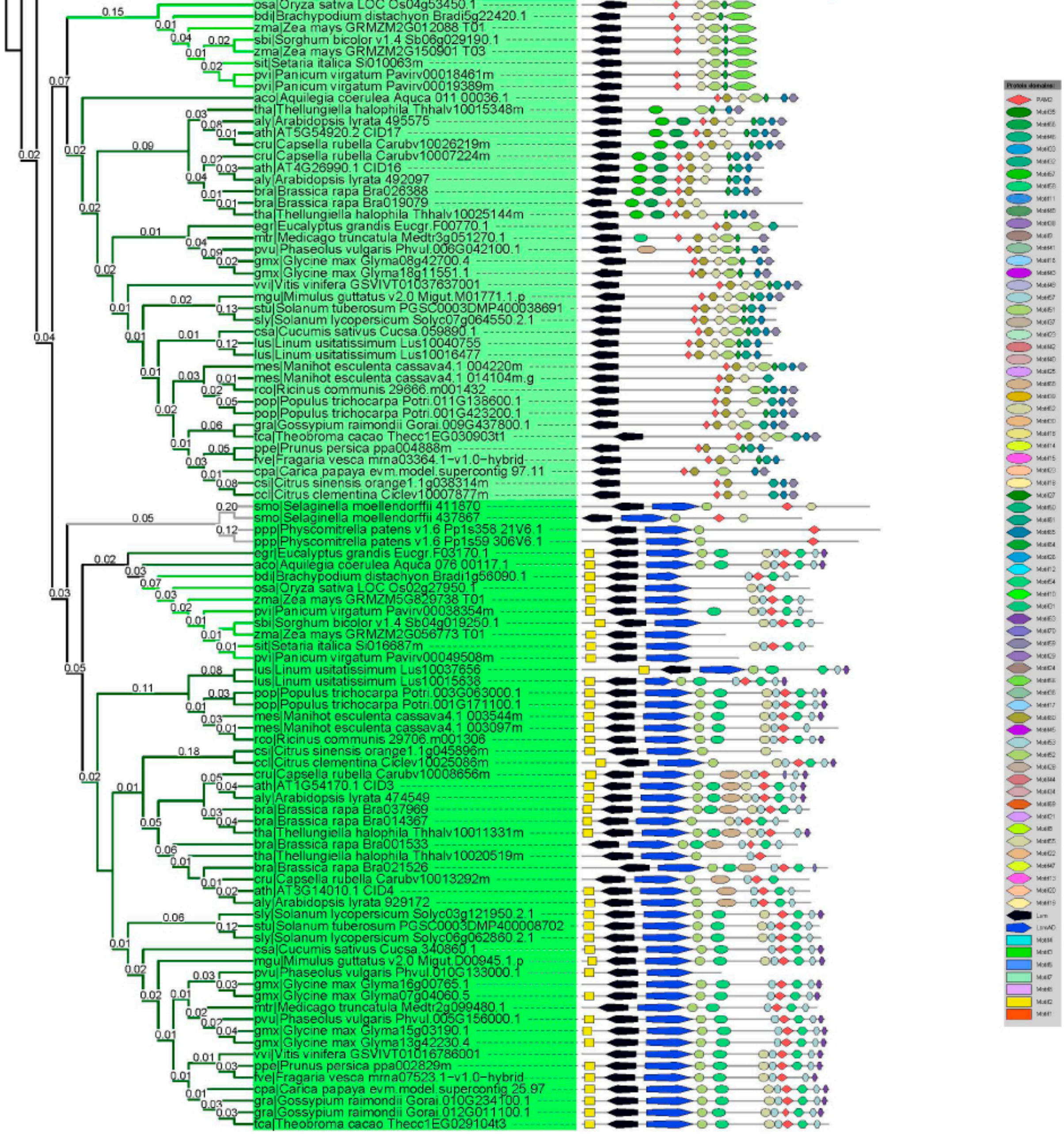

Additional file 4. Phylogenetic tree of Ataxin-2 proteins based on Lsm domain. The topology were generated by the NJ method; statistical significance in percentages above 50% for NJ. The domain architecture based on sequence logos is depicted next to the phylogenetic tree.
