# Supplementary material for: Insights into the evolution and domain structure of ataxin-2 proteins across eukaryotes
Source: BMC Res Notes. 2014 Jul 15;7:453. doi: 10.1186/1756-0500-7-453 (PMC4105795; doi:10.1186/1756-0500-7-453)
Supplement: Additional file 5 — Catalog of 71 sequence logos generated from 216 Ataxin-2 proteins. [file 1756-0500-7-453-S5.pdf]

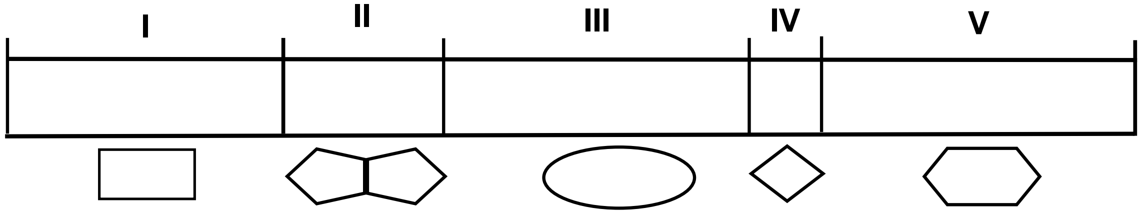

Logo Number	Symbol / Sequence
[1]	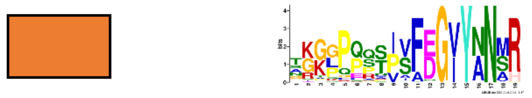
[2]	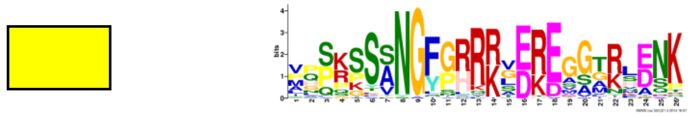
[3]	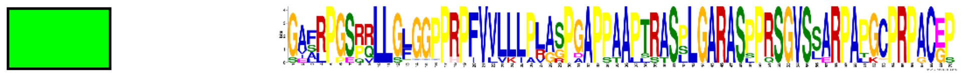
[4]	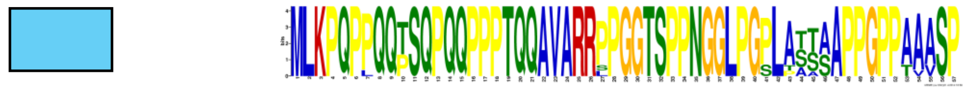
[5]	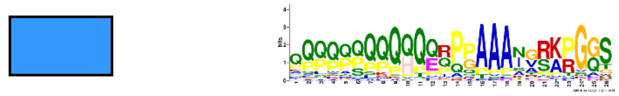
[6]	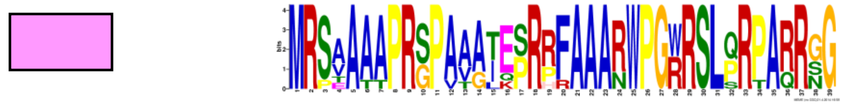
[7]	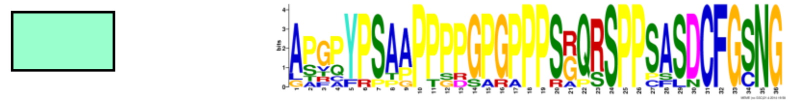
[9]	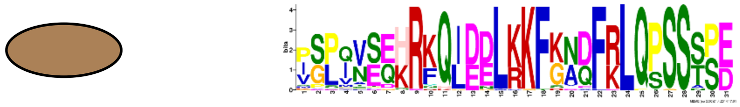
[11]	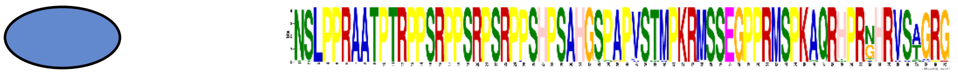
[14]	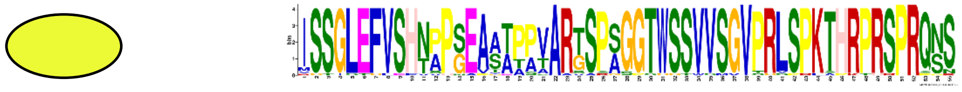
[15]	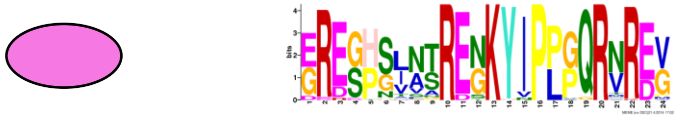

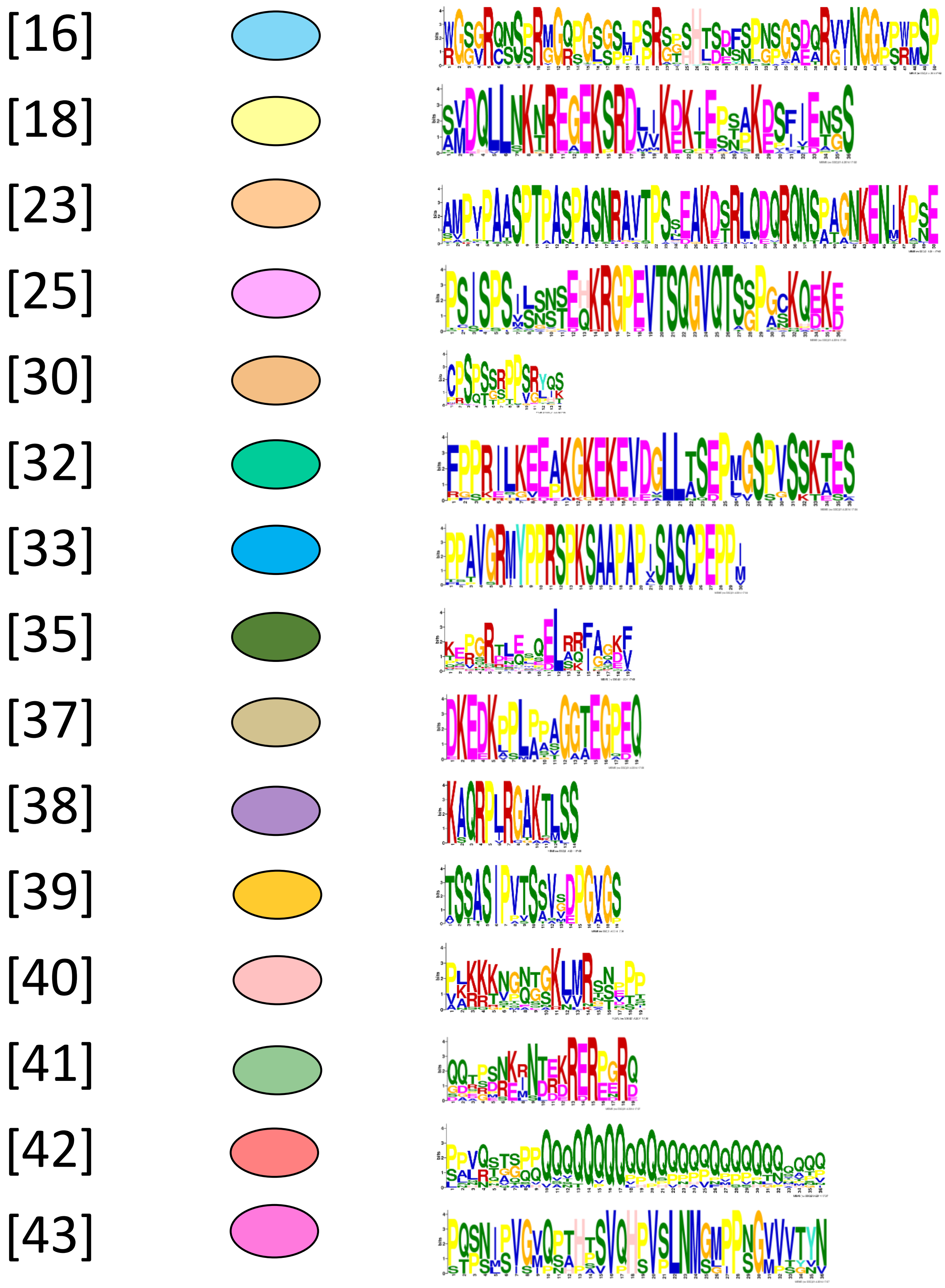

[16]
[18]
[23]
[25]
[30]
[32]
[33]
[35]
[37]
[38]
[39]
[40]
[41]
[42]
[43]

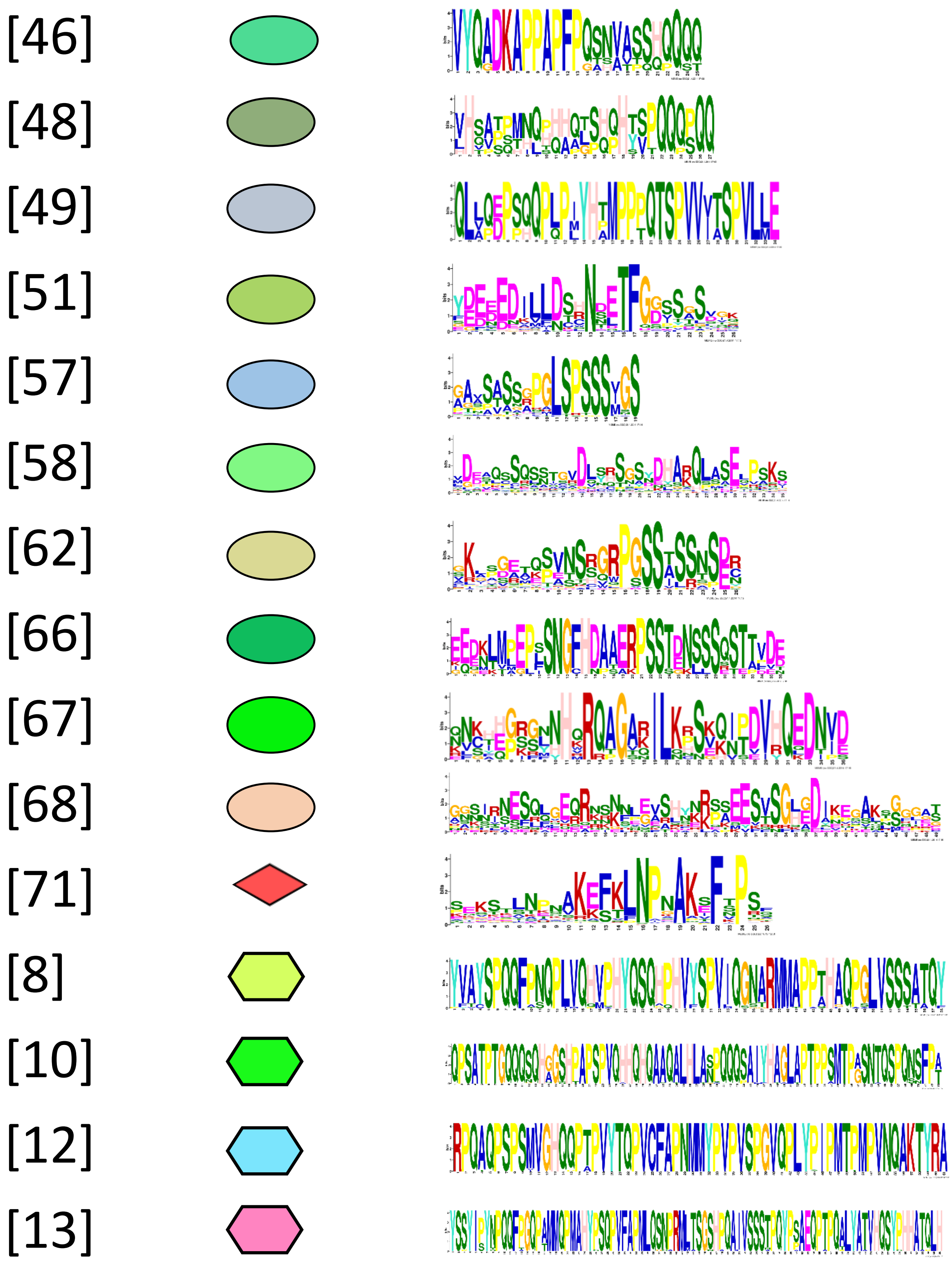
[46]
[48]
[49]
[51]
[57]
[58]
[62]
[66]
[67]
[68]
[71]
[8]
[10]
[12]
[13]

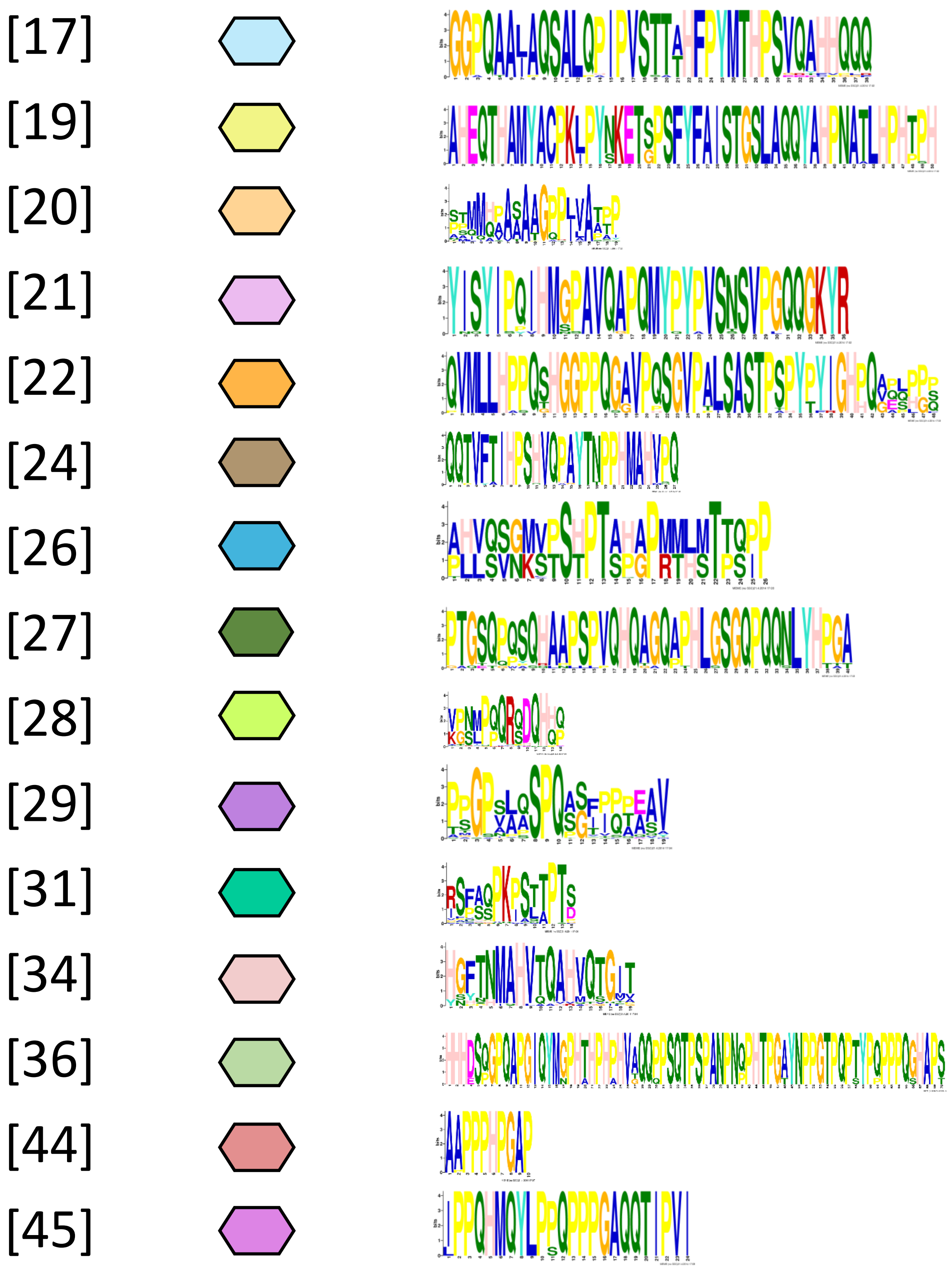
[17]
[19]
[20]
[21]
[22]
[24]
[26]
[27]
[28]
[29]
[31]
[34]
[36]
[44]
[45]

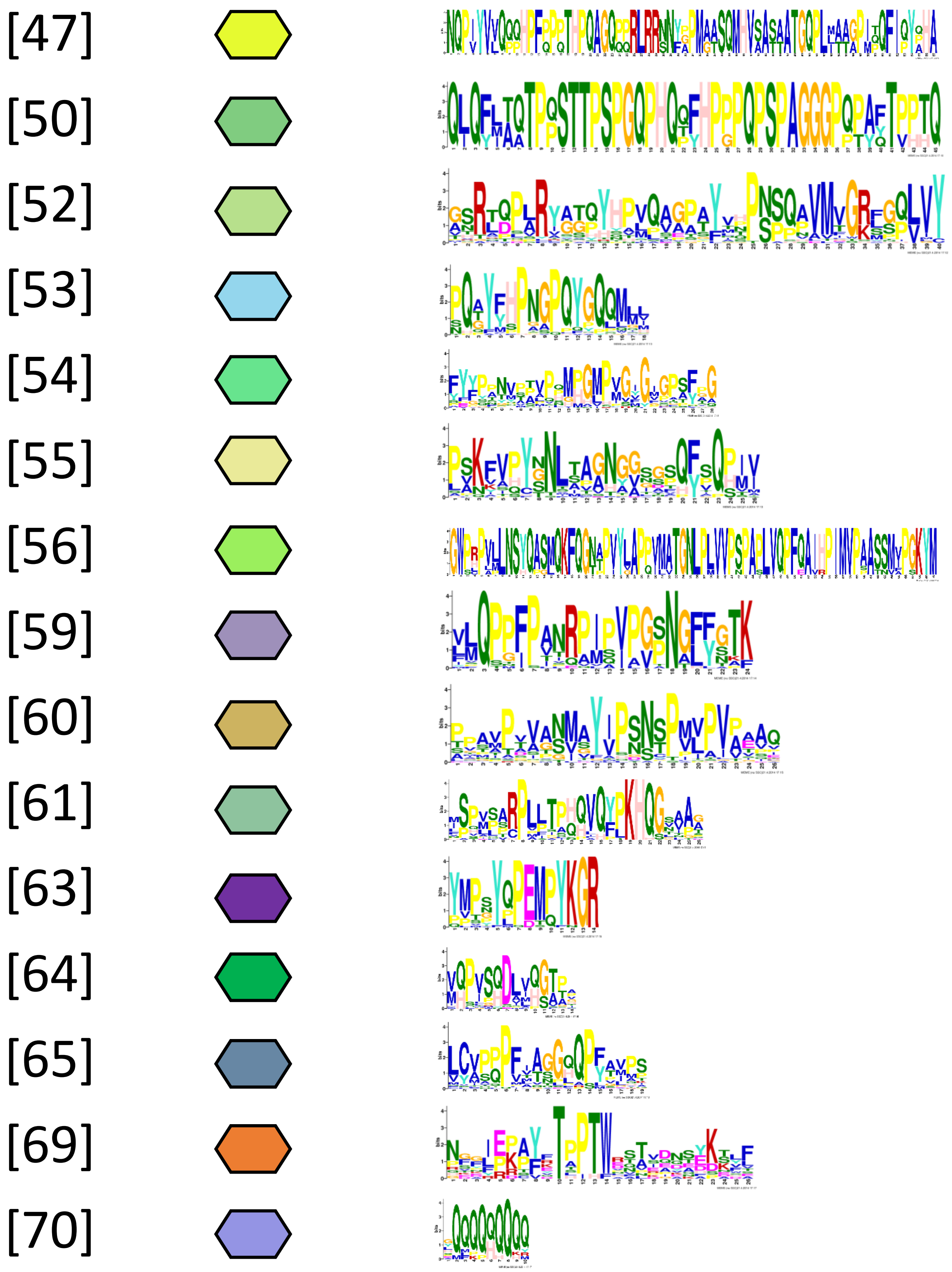

Additional file 5. Catalog of 71 sequence logos generated from 216 Ataxin-2 proteins.
