# Supplementary material for: Insights into the evolution and domain structure of ataxin-2 proteins across eukaryotes
Source: BMC Res Notes. 2014 Jul 15;7:453. doi: 10.1186/1756-0500-7-453 (PMC4105795; doi:10.1186/1756-0500-7-453)
Supplement: Additional file 6 — Alignment of the Ataxin-2 proteins from mammals. ClustalX 2.0.12 was used for sequence alignment and a default color code was applied. The location of regions encompassing sequence logos are enclosed by red rectangles and the Lsm, LsmAD and PAM2 domains by black rectangles. [file 1756-0500-7-453-S6.pdf]

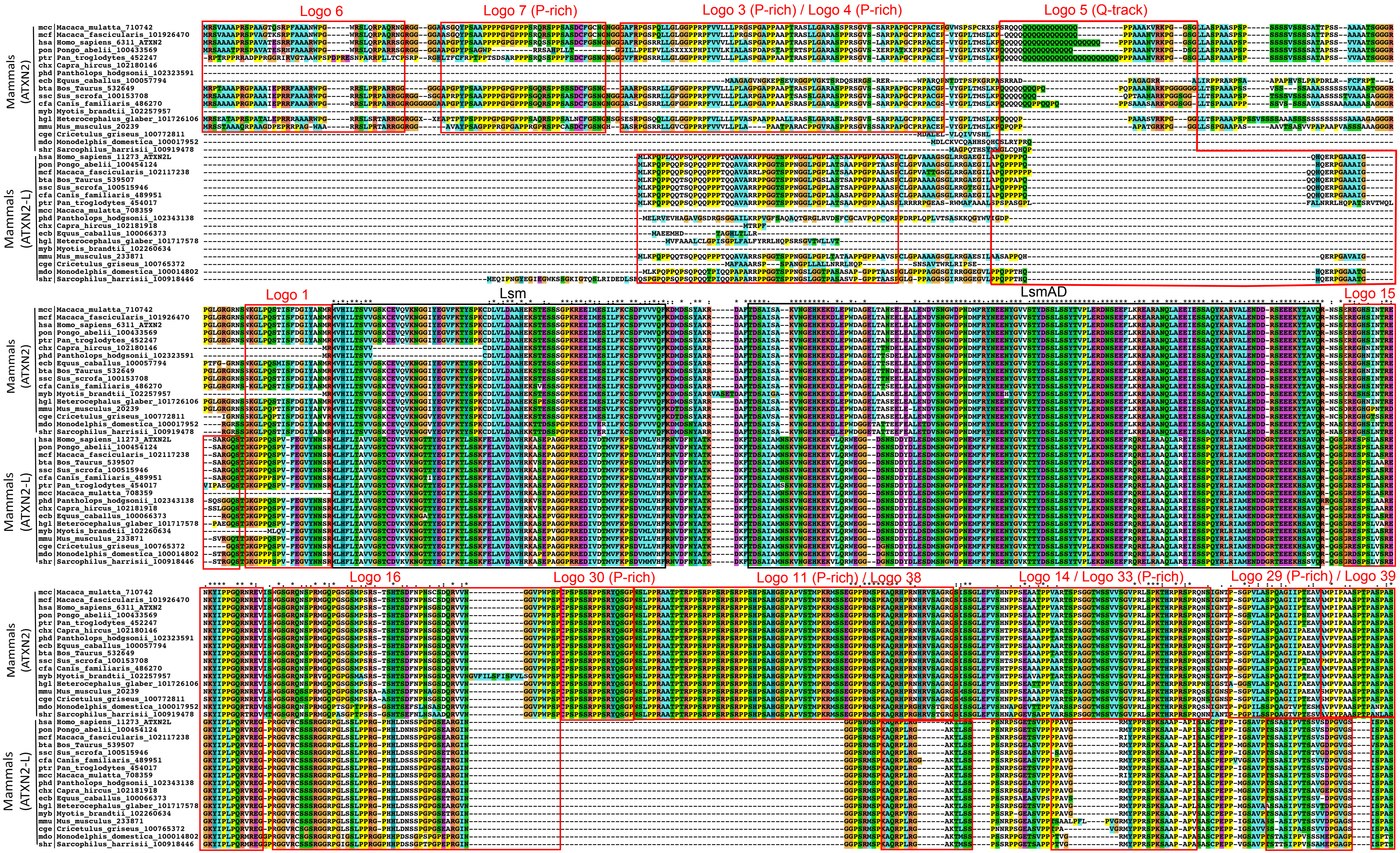

Logo 6
Logo 7 (P-rich)
Logo 3 (P-rich) / Logo 4 (P-rich)
Logo 5 (Q-track)
Mammals (ATXN2)
Mammals (ATXN2-L)
Logo 1
Lsm
LsmAD
Logo 15
Logo 16
Logo 30 (P-rich)
Logo 11 (P-rich) / Logo 38
Logo 14 / Logo 33 (P-rich)
Logo 29 (P-rich) / Logo 39

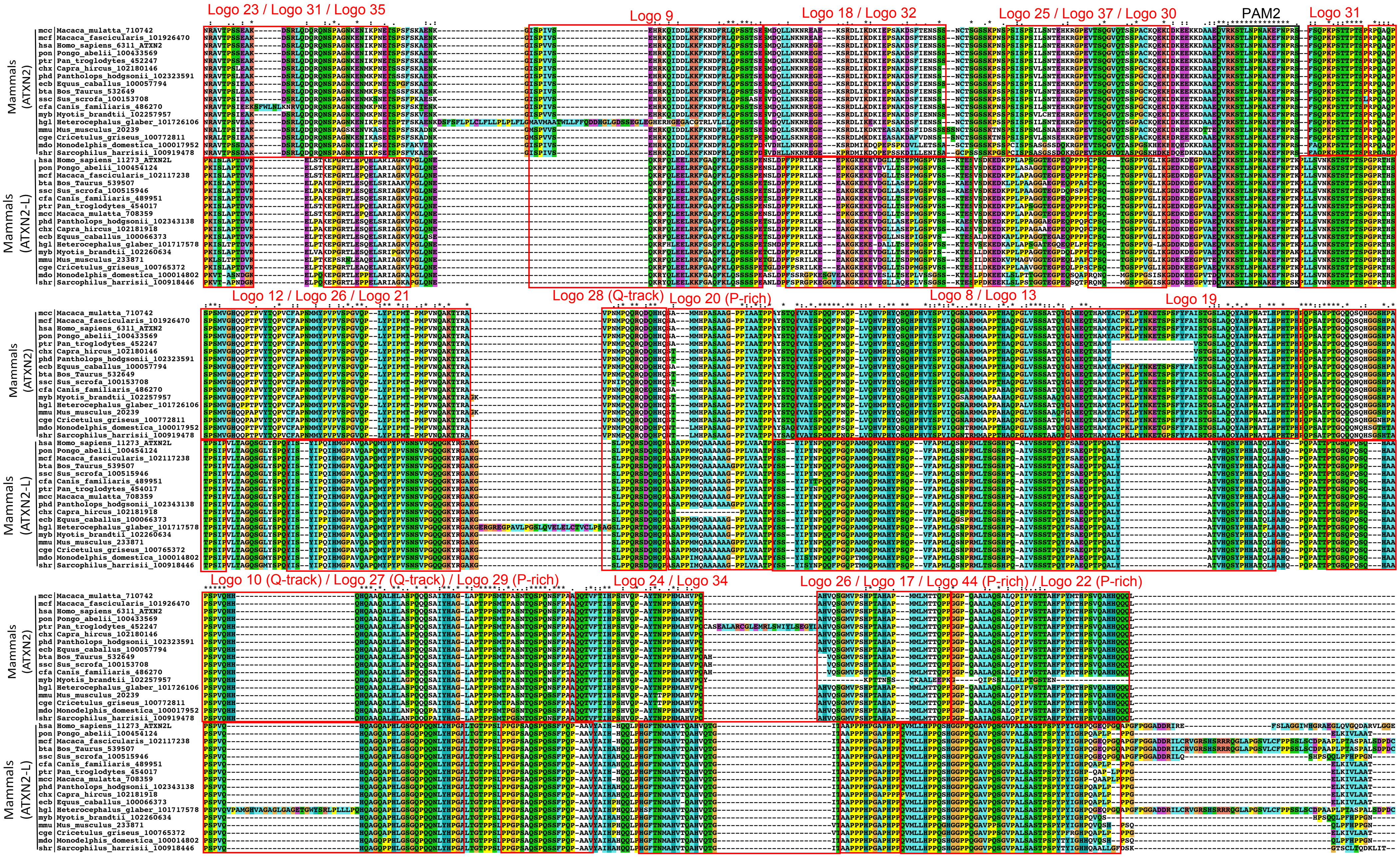

Additional file 6. Alignment of the Ataxin-2 proteins from mammals. ClustalX 2.0.12 was used for sequence alignment and a default color code was applied. The location of regions encompassing sequence logos are enclosed by red rectangles and the Lsm, LsmAD and PAM2 domains by black rectangles.
