# Supplementary figures and images for: Insights into the evolution and domain structure of ataxin-2 proteins across eukaryotes
Source: BMC Res Notes. 2014 Jul 15;7:453. doi: 10.1186/1756-0500-7-453 (PMC4105795; doi:10.1186/1756-0500-7-453)

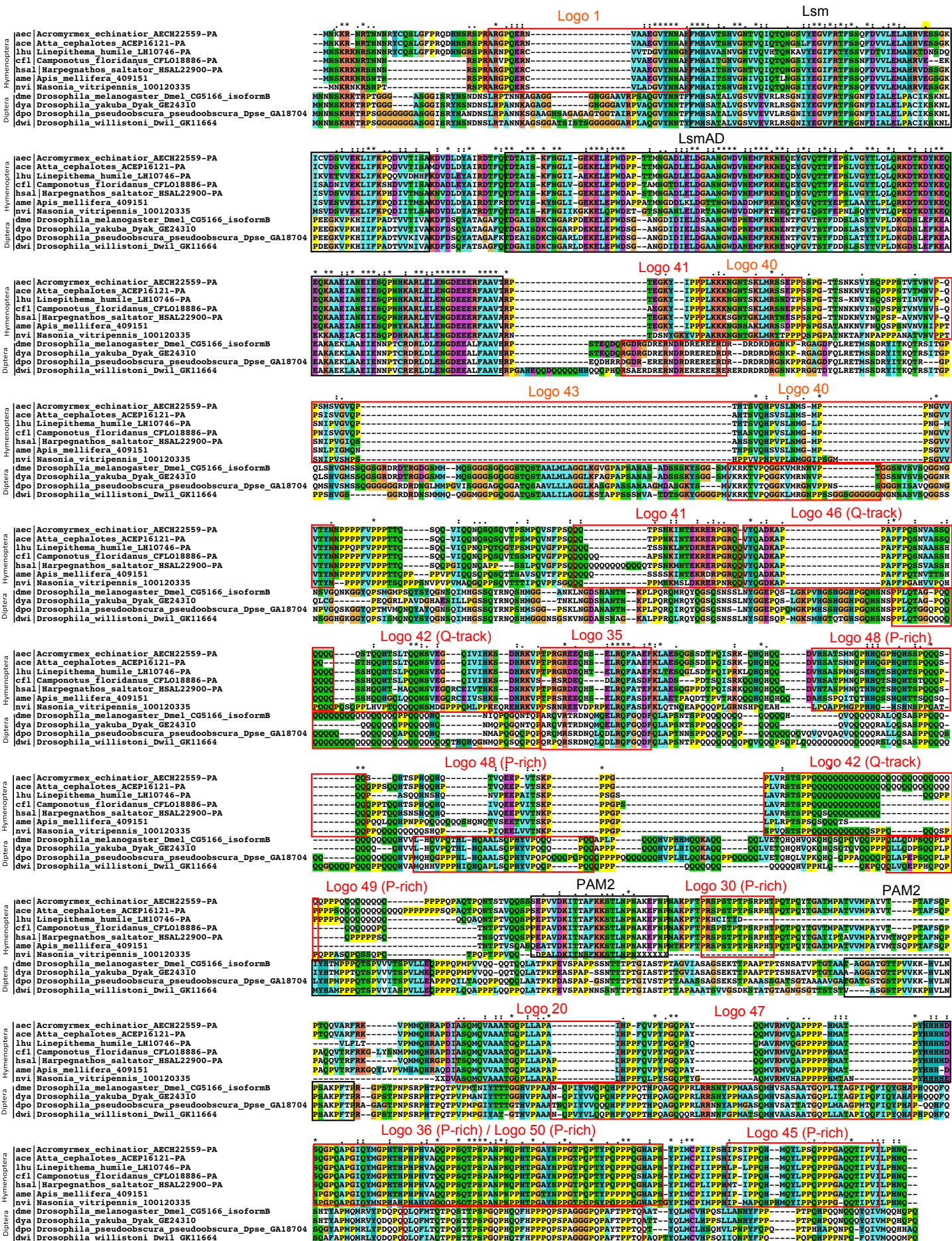

Supplement: Additional file 7 — Alignment of the Ataxin-2 proteins from insects (Orders Hymenoptera and Diptera). The sequence alignments were performed as described in Additional file 6. [file 1756-0500-7-453-S7.pdf]
