# Supplementary material for: Insights into the evolution and domain structure of ataxin-2 proteins across eukaryotes
Source: BMC Res Notes. 2014 Jul 15;7:453. doi: 10.1186/1756-0500-7-453 (PMC4105795; doi:10.1186/1756-0500-7-453)
Supplement: Additional file 8 — Alignment of the Ataxin-2 proteins from angiosperms. The sequence alignments were performed as described in Additional file 6. [file 1756-0500-7-453-S8.pdf]

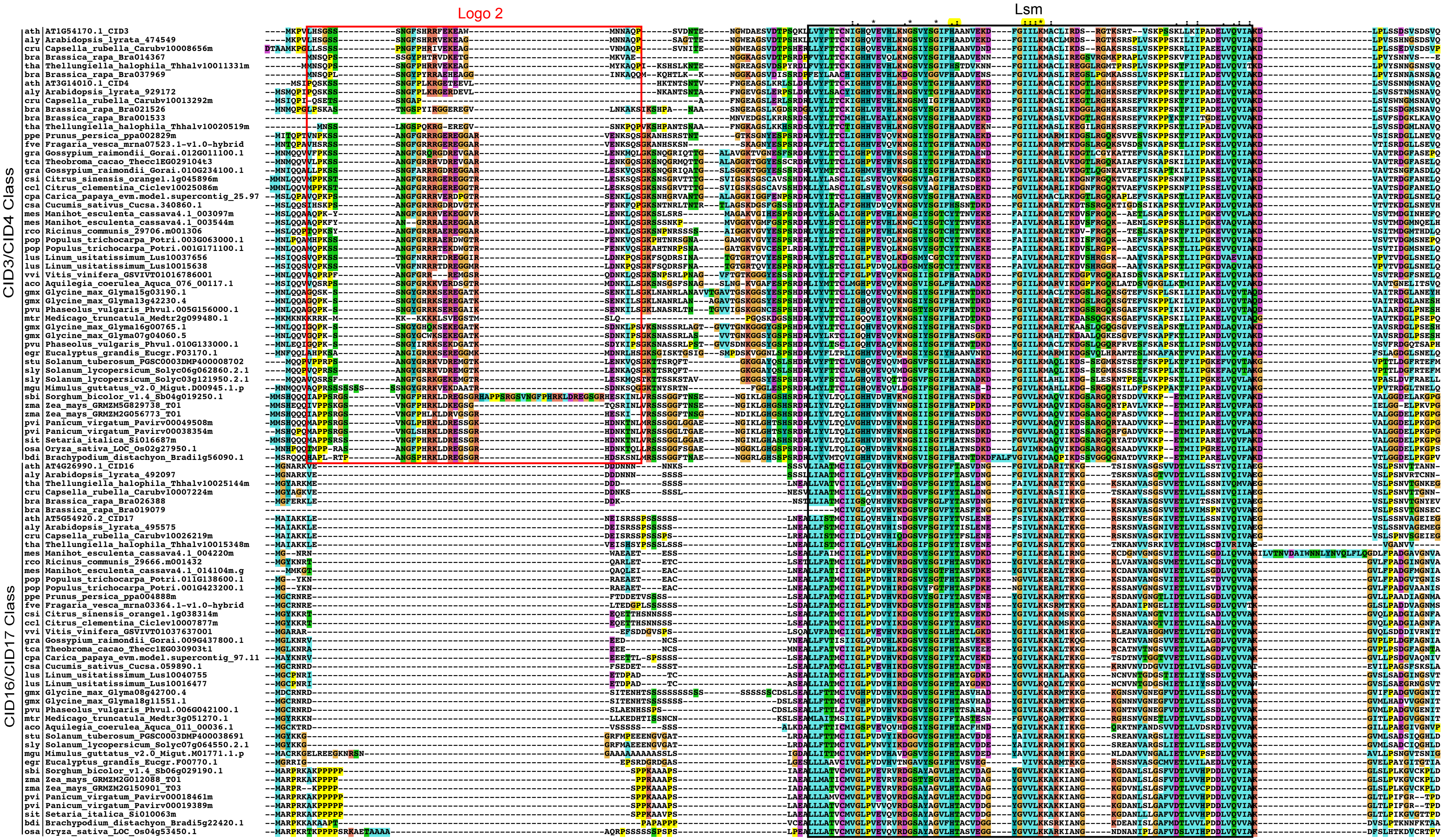
Logo 2
Lsm
CID3/CID4 Class
CID16/CID17 Class

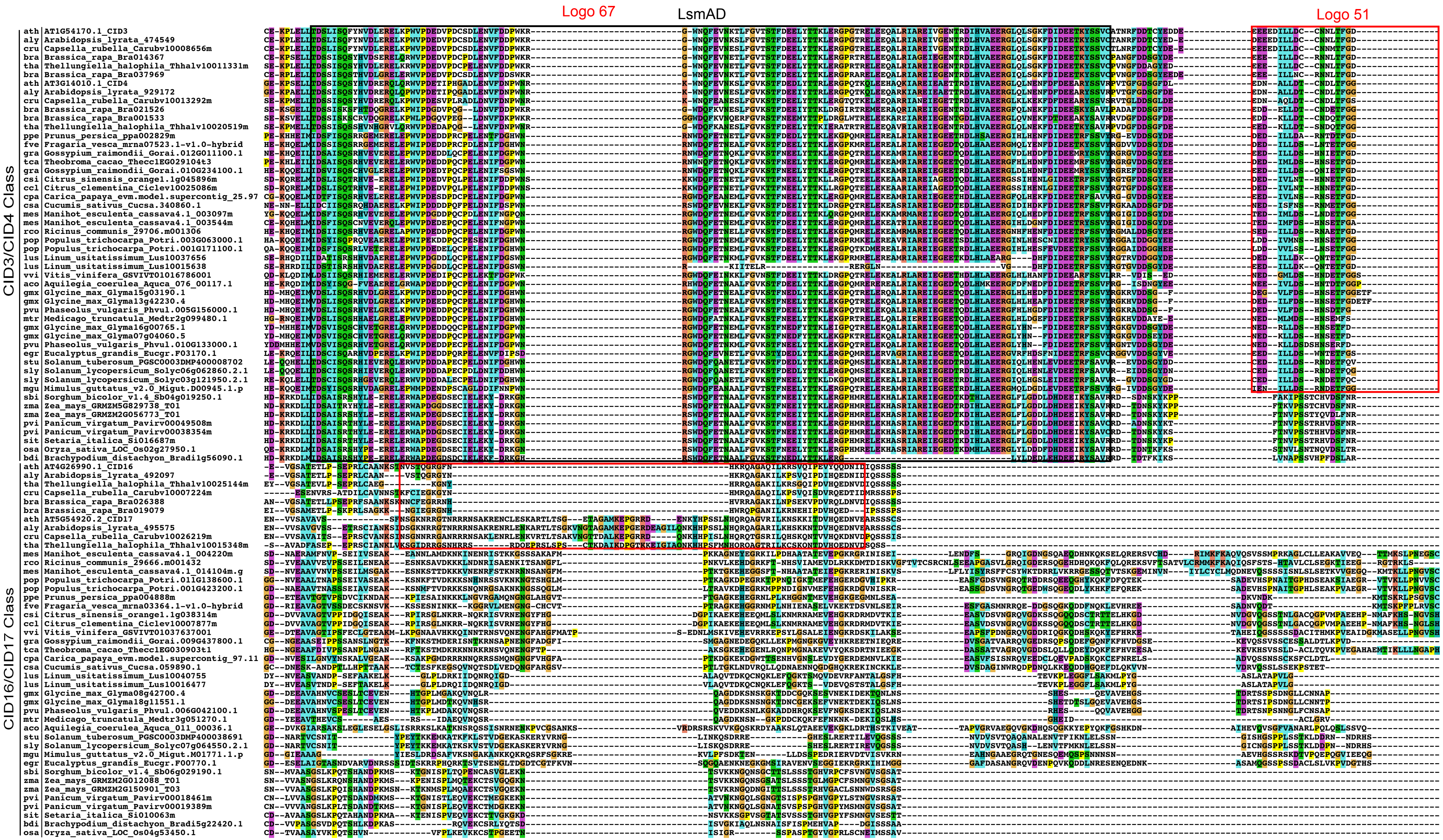

Logo 67
LsmAD
Logo 51
CID3/CID4 Class
CID16/CID17 Class

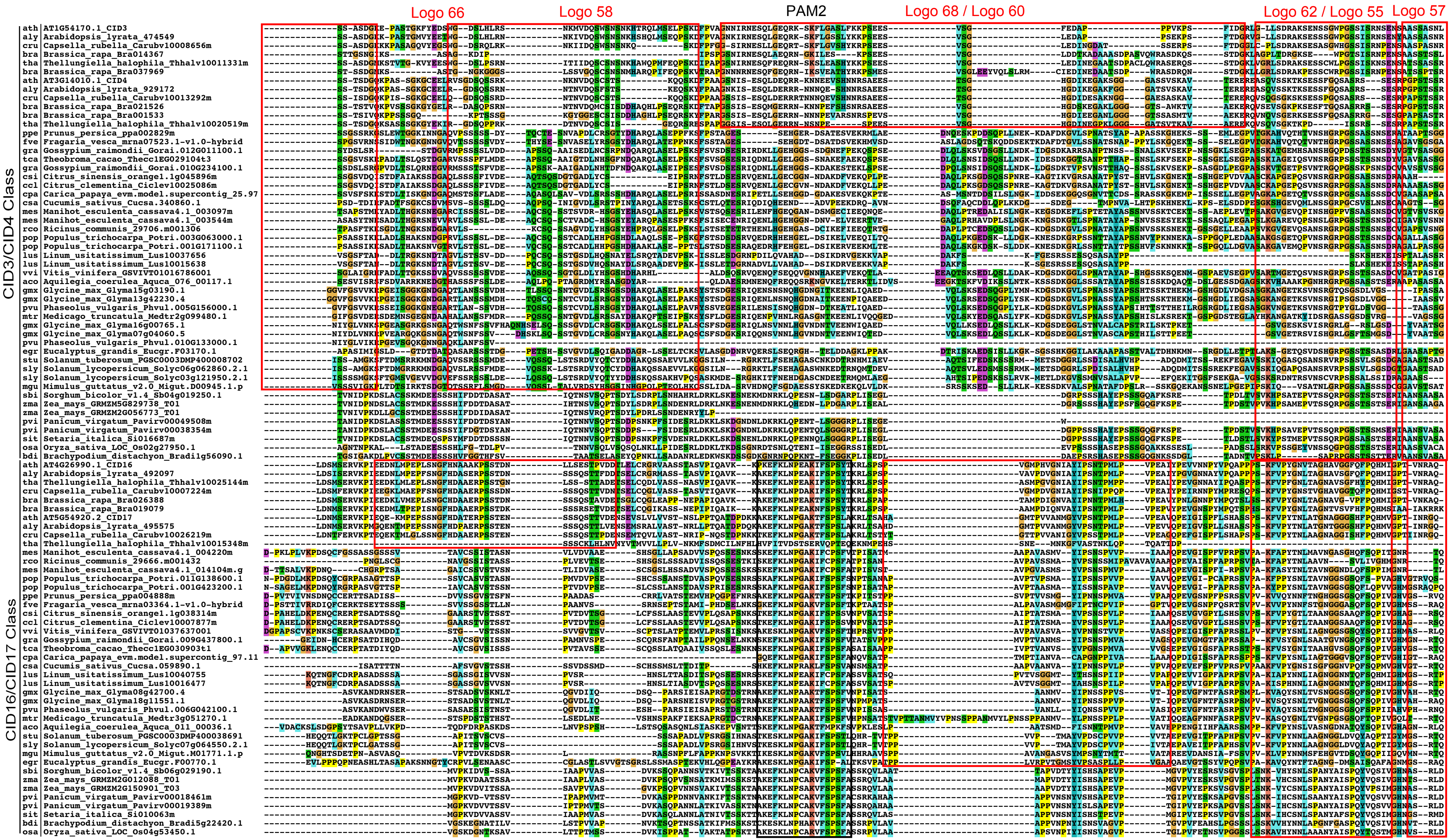
Logo 66
Logo 58
PAM2
Logo 68 / Logo 60
Logo 62 / Logo 55
Logo 57
CID3/CID4 Class
CID16/CID17 Class

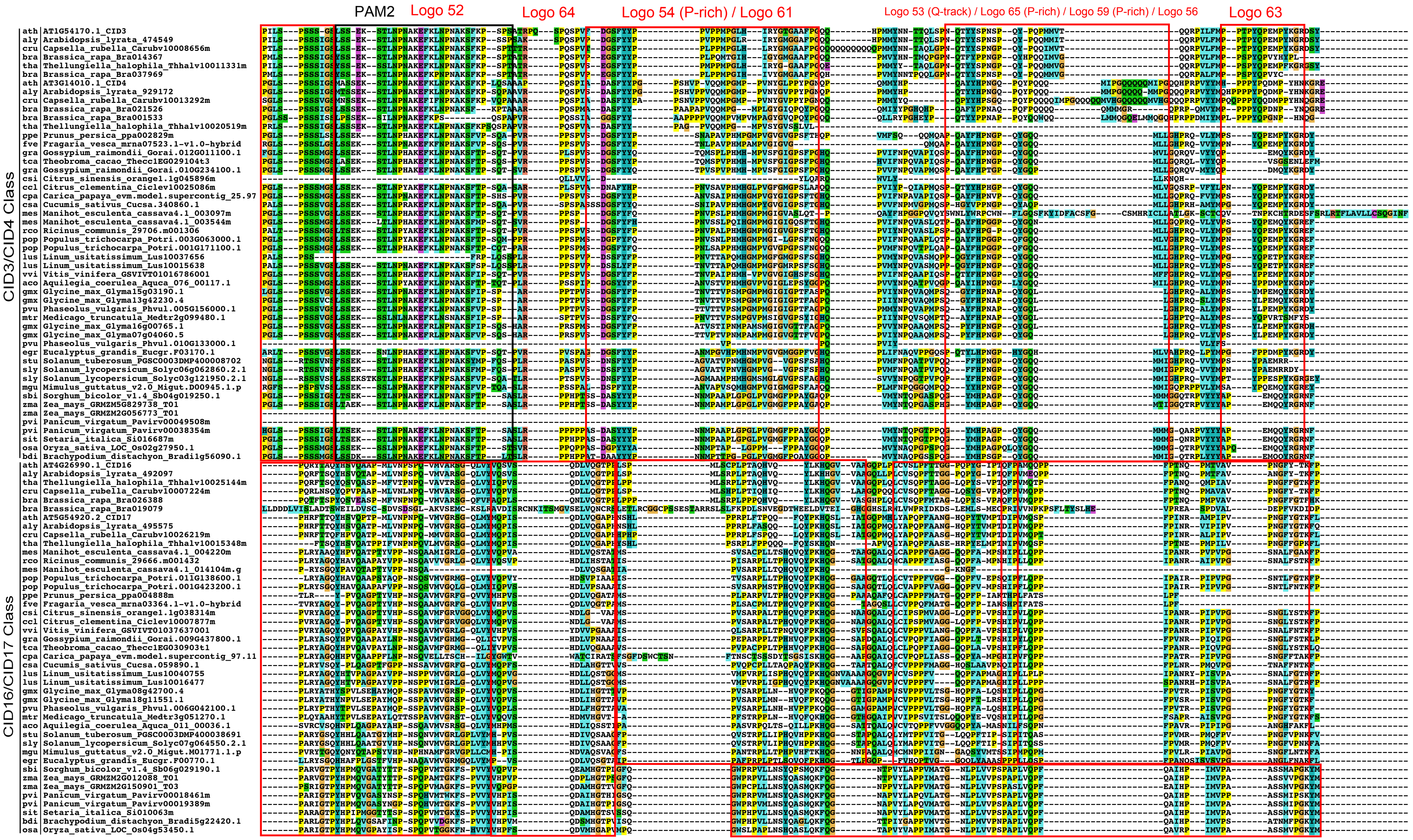

Additional file 8. Alignment of the Ataxin-2 proteins from angiosperms. The sequence alignments were performed as described in Additional file 6.
