# Supplementary material for: Insights into the evolution and domain structure of ataxin-2 proteins across eukaryotes
Source: BMC Res Notes. 2014 Jul 15;7:453. doi: 10.1186/1756-0500-7-453 (PMC4105795; doi:10.1186/1756-0500-7-453)
Supplement: Additional file 2 — Protein sequence alignment of the CID3, CID4, CID16 and CID17. The sequence alignments were performed using ClustalX 2.0.12; default colors were used. Regions encompassing sequence Lsm, LsmAD and PAM2 motifs are enclosed by rectangles. [file 1756-0500-7-453-S2.pdf]

```
AtCID16  ---------------------------MGNARKVEDDDNNNNNKSSSS----VLIAATMCIIGLQVHVHVKDGSVFS
AtCID17  ---------------------------MAIAKKLENEISRSSPSSSSSLNEALLISTMCIIGLQVHVHINDGSVFS
AtCID3   MKPVLHSGSSSNGFSHRRFEKEAWMNNAQPSVDNTENGWDAESVDTPSQKLLVYFTTCNIGHQVEVHLKNGSVYS
AtCID4   -MSIPQSKSSSNGFPLKRGETEEVLHKTN--TSNTVFNGEAGSLKRLSLDRLVYFTTCKIGHHVEVHLRNGSVYT

                                              Lsm
AtCID16  GIFFTASVDNGFGIVLKDARITKKG-----TSISNVASGSVVDTLVILSSTIVQIIAEGVSLPSNVT--------
AtCID17  GIFYTVSLENEFSIVLKNAKLTKKG-----RSKSNVESGKIVETLVILSSNIVQIVAEGVSLSSNVAGEIEGENV
AtCID3   GIFHAANVEKDFGIILKMACLIRD--SRGTKSRT--VSKPSSKLLKIPADELVQVIAKDLPLSSDSVSDSVQCEK
AtCID4   GIFHAANVEKDFGIILKMACLIKDGTLRGHKSRSEFVRKPPSKTFIIPADELVQVIAKDLSVSSNNMSNAVQGEK

                                                                      LsmAD
AtCID16  ----------TANNEVGSATETLPSEPRL----CAANKSTNVST---------QGR---------------GFNHK
AtCID17  VSAVAVSSFNSGKNRRGTNRRRNSAKREN----CLESKARTLTSGETAGAMKEPGRRDEVGILQNKYHPSSLNHQ
AtCID3   PLELLTDSLISQFYNVDLERELKPWVPDEDVPDCSDLENVFDDPWKRGWNQFEVNKTLFGVTSTFDEELYTTKLE
AtCID4   PSELLTDSSISQSYHVDRERQLQRWVPDETIPHGADLENVFDNPWNRKWNQFEVNKSLFGVKSTFDEDLYTTRLE

AtCID16  RQAGAQILKRSVQ--IPEVYQQDNIDIQSSSSSLDSMSERVKPIEEDNLMPEPLSNGFHNAAAK-----------
AtCID17  RQAGVRILKNSKK--ITDVHQEDNVEARSSSCSLDNMSERVKPIEQE-KMPEPSSNGFHDATER-----------
AtCID3   RGPGTRELEEQALRIAREIVGENTRDIHVAEERGLQLSGKFDIDEETKYSSVCATNRFDDTCYEDDEEEEDILL
AtCID4   RGPQTKQLEEHAQKIAREIEAETTRDIHVAEERGLQLNENFDFDEEARYSSVRPVTGFGDSGFD----LEDNALL

                                                                          PAM2
AtCID16  PSSTDNLLSESTPVDDTLELCRGRVAASS--TASVPIQAVKK---------------------PKEFKLNPEAKI
AtCID17  PSSTENSSSQSTTVDENSEVSLVLVVSTNSLPPTQATDPDKK---------------------AKEFKLNPGAKT
AtCID3   DCCNNLTFGDSSASDGKEPASTGKFYEDS--WGDSLHLRSNKMVDQSWSNSNKHTRQLMSELPSKDFPVAGNNIR
AtCID4   DTCNDLTFGGSSTSDGQKPASSGKGCEELRVSGDSQSSRKNKNVDQSCSTSKQQ----------SKDFPAAGSNIS

AtCID16  FSPSYIKRLSPSPVGM-PHVGNIAYIPSNTPMLPVPEAIYP------------EVVNNPYVPQAPPPSKFVPYGN
AtCID17  FSPSLAKRLTSAHAGMTPVVANMGYVPSNTPMLPVPEAVQP------------EIGISPFLSHASSPSKFVPYTN
AtCID3   NESQLGEQRKSKFLGASLFKKPSEESVSGFEDAPPPVKPS--------FIDGRLGLLSDRAKSENSSGWPGSSIS
AtCID4   -ESQLDEQRRKNNEEVSHNNRSAEESTSGHGDIKEGAKSGGGASSVSKAVTEREREASQVSSKTKSESSFGQSAS

                                     PAM2
AtCID16  VTAGHAVGGFQFPQHMIGPTVNRAQPQRYTAQYHSVQAAPMLVNPSPQVMVARSGQLVYVQSVSQDLVQGTPPLS
AtCID17  LATGNAGGGSHFPQHMVGPTINRGQPHRFTTQYHSVQPTPMLVNPNPQVMVGRSGQLMYMQPISQDLVQGAPHNS
AtCID3   RNSENSAASSASNLPILSPSSS-GSLSS-EKSTLNPNAKEFKLNPNAKSFKP-SPSATRPQSPQSPVFDGSFYYP
AtCID4   RSSESRPGPSTSSRPGLSPSSSIGSMASSEKSTLNPNAKEFKLNPKAKSFKP-LQSAAAP--PQSPIADASFYYP

AtCID16  PMLSCPLPTAQHVQYLKHQGVVAAG-QPLPLCVSLPFTTGGPQPYG-IPTQFPAMQQPPFPTNQ---PMTVAVPN
AtCID17  HLPPRPLFTPQQFQYPKHQSLIATG-QPMHLYAPQPFAANGHQPYTVMPTDIPVMQSP-FPINR---AMPIPVPN
AtCID3   -----PVPPMPGLHIRYGTGAAFPGQQHPMMYNNTTQLSPNQTYYSPNSPQYP---QPMMVTQQ---RPILFMPP
AtCID4   GPSHVPVQQMPGMPPVNYGLPPYPGNQPQMMYHPQAYYHPNGQPQYPQQQMIPGQQQQQMIPGQQHPRPVYYMHP

AtCID16  GFY-TKFP-------
AtCID17  GFYGTKFL-------
AtCID3   TPYQPEMPYKGRDSY
AtCID4   PPYPQDMPYHNKGRE
```

Additional file 2. Protein sequence alignment of the CID3, CID4, CID16 and CID17. The sequence alignments were performed using ClustalX 2.0.12; default colors were used. Regions encompassing sequence Lsm, LsmAD and PAM2 motifs are enclosed by rectangles.
